# Distinct Circulating Expression Profiles of Long Noncoding RNAs in Heart Failure Patients With Ischemic and Nonischemic Dilated Cardiomyopathy

**DOI:** 10.3389/fgene.2019.01116

**Published:** 2019-11-12

**Authors:** Fang Lin, Xin Gong, Ping Yu, Aixue Yue, Qingshu Meng, Liang Zheng, Tian Chen, Lu Han, Hao Cao, Jianhong Cao, Xiaoting Liang, Hao Hu, Yuan Li, Zhongmin Liu, Xiaohui Zhou, Huimin Fan

**Affiliations:** ^1^Research Center for Translational Medicine, Shanghai East Hospital, Tongji University School of Medicine, Shanghai, China; ^2^Shanghai Heart Failure Research Center, Shanghai East Hospital, Tongji University School of Medicine, Shanghai, China; ^3^Institute of Integrated Traditional Chinese and Western Medicine for Cardiovascular Chronic Diseases, Tongji University School of Medicine, Shanghai, China; ^4^Department of Heart Failure, Shanghai East Hospital, Tongji University School of Medicine, Shanghai, China; ^5^Department of Ultrasound, Shanghai East Hospital, Tongji University School of Medicine, Shanghai, China; ^6^Department of Cardiothoracic Surgery, Shanghai East Hospital, Tongji University School of Medicine, Shanghai, China; ^7^Translational Medical Center for Stem Cell Therapy, Shanghai East Hospital, Tongji University School of Medicine, Shanghai, China

**Keywords:** dilated cardiomyopathy, ischemic cardiomyopathy, long noncoding RNA, messenger RNA, microarray, expression profile

## Abstract

Ischemic cardiomyopathy (ICM) and dilated cardiomyopathy (DCM), with distinct long-term prognosis and responses to treatment, are two major problems that lead to heart failure (HF) ultimately. In this study, we investigated the long noncoding RNA (lncRNA) and messenger RNA (mRNA) expressions in the plasma of patients with DCM and ICM and analyzed the different lncRNA profile between the two groups. The microarray analysis identified 3,222 and 1,911 significantly differentially expressed lncRNAs and mRNAs between DCM and ICM group. The most enriched upregulated functional terms included positive regulation of I-kappaB kinase/nuclear factor-kappaB signaling and regulation of cellular localization, while the top 10 downregulated genes mainly consisted of acid secretion and myosin heavy chain binding. Furthermore, the Kyoto Encyclopedia of Genes and Genomes pathway analysis revealed that the differentially expressed lncRNA-coexpressed mRNAs between DCM and ICM group were significantly enriched in the natural killer cell mediated cytotoxicity and ras signaling pathway respectively. Quantitative real-time PCR confirmed 8 of 12 lncRNAs were upregulated in DCM group compared to ICM group which was consistent with the initial microarray results. The lncRNA/mRNA coexpression network indicated the possible functions of the validated lncRNAs. These findings revealed for the first time the specific expression pattern of both protein-coding RNAs and lncRNAs in plasma of HF patients due to DCM and ICM which may provide some important evidence to conveniently identify the etiology of myocardial dysfunctions and help to explore a better strategy for future HF prognosis evaluation.

## Introduction

Chronic heart failure (CHF), a clinical syndrome that results from various cardiovascular diseases, represents one of the largest contributors to healthcare burden and mortality worldwide and remains a worsening global problem especially in aging populations (Kumarswamy and Thum, 2013). As a heterogeneous entity, recent advances of heart failure (HF) treatment can slow down its progression to some extent. In-depth understanding of novel pathological mechanisms is essential to develop effective therapy and assessment of HF prognosis in individual patients.

In response to various stress, compensatory cardiac remodeling characterized by progressive cardiac hypertrophy and fibrosis occurred and ultimately lead to cardiac dysfunction known as HF. The etiology of CHF has commonly been categorized into ischemic cardiomyopathy (ICM) and non-ICM, distinguished by the presence or absence of significant coronary artery disease (Felker et al., 2000). In addition, these two categories have distinct long-term prognosis (Felker et al., 2000) and different responses to treatment (Follath et al., 1998). Recent advances in the treatment and diagnosis of myocardial infarction (MI) have improved the life quality for MI patients, however, its mortality is still high since the occurring of severe complications, especially ventricular fibrillation, cardiac rupture, and eventually progressive HF. Nonischemic dilated cardiomyopathy (DCM), characterized by a progressive left ventricular (LV) chamber dilatation and global myocardial dysfunction, represents the most frequent subtype of non-ICM. However, the mechanism of DCM remains elusive and novel pharmacologic approaches to treat DCM scarce (Armstrong, 2006).

With the advances in recent technologies applied to medical diagnosis and treatment, new possibilities have emerged to understand the pathophysiology of many diseases. Recently, it is reported that noncoding RNAs, especially microRNAs (miRNAs), long noncoding RNAs (lncRNA), and circular RNAs, serve as epigenetic regulators of cardiac gene expression and thus significantly influence cardiac homeostasis and functions (Di Salvo, 2015). lncRNA, as a subclass of noncoding RNAs with a length of more than 200 nt and no open reading frame are profoundly involved in multiple cardiovascular diseases (Dangwal et al., 2016). A large-scale case−control association study identified the lncRNA MI-associated transcript as a risk allele for MI in Japanese subjects (Ishii et al., 2006). Recent report showed the association of polymorphisms in long noncoding RNA H19 with coronary artery disease risk in a Chinese population (Gao et al., 2015). Moreover, many results revealed the correlation of H19 expression with tumors (Matouk et al., 2015), vascular injury (Kim et al., 1994), atherosclerotic plaques (Han et al., 1996), and high blood pressure (Tragante et al., 2014).

Results from RNA deep sequencing revealed distinguished lncRNAs expression signature, but not messenger RNAs (mRNAs) or miRNAs in human LV between ischemic and nonischemic failing hearts, suggesting that lncRNA expression profiles are more sensitive to different HF etiologies than mRNA or miRNA expression (Yang et al., 2014). In addition, patients subjected to MI showed distinct lncRNAs expressions in blood cells from the control (Vausort et al., 2014). Recent report found that lncRNA LIPCAR should be a novel biomarker of cardiac remodeling and to predict future death in patients with HF (Kumarswamy et al., 2014). Although latest reports have collectively described the vital roles of lncRNAs in cardiovascular biology and diseases, questions that whether they can be used as biomarkers to discriminate the etiology or determine the efficacy of therapy of HF still exist.

In the present study, we compared the lncRNAs expression pattern through RNA microarray and performed multiple analyses to identify the possible functions and pathways of the differential expressed lncRNAs in plasma of patients with DCM and ICM. These results provided some important evidences which may help to conveniently identify the etiology involved in the reverse remodeling of heart with DCM or ICM, and possibly to dynamically determine the efficacy of therapy, and explore a better future prognosis evaluation.

## Materials and Methods

### Ethics Statement

The study conforms to the ethical guidelines of the 1975 Declaration of Helsinki. The Ethics Committee of Shanghai East Hospital, Tongji University School of Medicine approved the protocol of this study(Num: ECSEH2016-037) and all enrolled patients gave the written informed consent.

### Study Population and Blood Collection

Twenty peripheral fasting blood samples were donated by CHF patients with DCM (n = 11) and ICM (n = 9). Plasmas were analyzed by RNA microarray (Arraystar Human lncRNA microarray, v4.0, containing 40,173 lncRNAs and 20,730 coding transcripts). Inclusion criteria were as follows: (1) All patients had ventricular dilation and LV ejection fraction < 50%. (2) Patients with ICM were diagnosed by coronary angiogram or suffered from MI before. (3) All patients with DCM were proved nonischemic by coronary angiogram or coronary computed tomography angiography and in the absence of hypertensive, valvular, or congenital heart diseases. Patients with the following conditions were excluded from the study: (1) Cardiac resynchronization/modulation therapy in 3 months, (2) acute MI in 3 months, (3) coronary artery bypass grafting in 3 months, (4) malignant tumor or immune diseases were excluded.

Peripheral blood was collected in Vacutainer plastic blood collection tubes with spray-coated K2EDTA (Kehua Bio-Engineering co., catalog # 25022101, Shanghai, China). Plasma samples were collected within 4 h according to standard procedures and frozen at −80°C prior to the RNA extraction.

Echocardiograms were done with (Philips, IE33 Ultrasound, Netherlands). Pro-Brain Natriuretic Peptide were measured by Electrochemical Luminescence Automatic Immunoassay System(Roche, Cobas E411, Swiss).

### lncRNA and mRNA Microarray Experiment

Total RNA was prepared with TRIzol Reagent (Thermo Fisher Scientific, catalog # 15596026, Waltham, MA, USA) in according to the manufacturer’s instructions. The quality and quantity of extracted RNA were assessed by the NanoDrop ND1000 Spectrophotometer (Thermo Fisher Scientific, Waltham, MA, USA) (**Table S1**). RNA samples were labeled by Quick Amp Labeling Kit (Agilent, catalog # 5190-0442, Palo Alto, CA, USA), and purified by RNeasy Mini Kit (Qiagen, catalog # 74104, Dusseldorf, German). The microarray hybridization were performed using Agilent Gene Expression Hybridization Kit (Agilent, catalog # 5188-5242, Palo Alto, CA, USA). After washing, the hybridized arrays were scanned by Agilent DNA microarray Scanner (Agilent, G2505C, Palo Alto, CA, USA) and finally were analyzed by Agilent Feature Extraction software (Agilent, version 10.5.1.1, Palo Alto, CA, USA). The differentially expressed transcripts were identified by fold-change screening at a threshold more than two-fold and a *p*-value < 0.05.

### KEGG and GO Pathway Analysis

Statistical enrichment was evaluated by Kyoto Encyclopedia of Genes and Genomes (KEGG) and Gene Ontology (GO) pathway analysis. To explore the significant pathways of the differentially expressed genes in the KEGG database (http://www.genome.jp/kegg), pathway analysis was performed using DAVID software (https://david.ncifcrf.gov/). GO enrichment of the target genes was performed to elucidate the biological roles including molecular function, biological process, and cellular component aspects of the differentially expressed genes by the GOseq R package (http://www.geneontology.org) and corrected by *p*-value (*p* < 0.05 were considered significantly enriched).

### Chromosome Distribution of Differentially Expressed lncRNAs

According to the microarray analysis results in which 3,222 differentially expressed lncRNAs between DCM and ICM group were identified and their locations information provided in chromosomes. All the chromosome distribution of these lncRNAs were analyzed and presented in the histogram.

### Quantitative Real-Time PCR

lncRNA microarray data were validated using quantitative real-time PCR (qRT-PCR). SuperScript III Reverse Transcriptase (Thermo Fisher Scientific, catalog # 18080044, Waltham MA, USA) was used in accordance with the manufacturer’s instructions. qRT-PCR was performed using the Applied Biosystems ViiA 7 Real-Time PCR System (Thermo Fisher Scientific, Waltham MA, USA) and 2*PCR Master Mix (Arraystar Inc., catalog # AS-MR-006-5, Rockville, MD, USA). Primers were designed online using Primer 5 software and evaluated by the Basic Local Alignment Search Tool at the National Center for Biotechnology Information (**Table 1**). Cycle threshold (Ct) values were used to quantify the expression levels of genes as 2^−ΔΔCT^ according to previous report (Livak and Schmittgen, 2001). β-Actin was applied as a normalization control (Peng et al., 2017).

**Table 1:** Primers designed for quantitative real-time PCR (qRT-PCR) validation of candidate long noncoding RNAs (lncRNAs).

Name	Primers	Tm (°C)	Product length (bp)
ENST00000554552	F:5′ CAGAAGAAGGCAAGTCTCAATG 3′ R:5′ GAACAGTCCCAGAAAGATAGCA 3′	60	159
ENST00000494340	F:5′ CCAAAGTCCAGCTACCACAATA 3′ R:5′ TCCCGTGACCATAGGAAGATA 3′	60	172
NR_046647	F:5′ CAGGTCTACAGAGCAATGGTG 3′ R:5′ GCAAATTGAGTCTTCTGTTTTG 3′	60	77
NR_109994	F:5′ GCCAGATAAGAATAGCTCCAGT 3′ R:5′ TAGCAGTGAGCAAGACTCCATA 3′	60	163
NR_029376	F:5′ CCAACCCGATACTATACACGAC 3′ R:5′ GGTCTTACCGTTGCTGCTTAT 3′	60	72
T109935	F:5′ TTGCCGATGTGGAGTGATT 3′ R:5′ TTGTCTGGTCCTTTGCTTGA 3′	60	156
T023556	F:5′ CGTATGCCCCTAGTCCTGTT 3′ R:5′ CACGGAAGTTGAGTCTGTGGTT 3′	60	182
T201134	F:5′ ACTGGAACTACGGAAATGACG 3′ R:5′ GATGTTGCGAGTTGTCTTCAC 3′	60	102
T226011	F:5′ TCCAATCAACCGCCTTCTC 3′ R:5′ TAACCCTATGGCCCTCCTT 3′	60	257
T207073	F:5′ GGCATTTCTTTAGCTGTTGC 3′ R:5′ TCTTCTTTATGGAGGAGGTGAC 3′	60	192
T191270	F:5′ ACGGCTAAAATCCAAAGGC 3′ R:5′ TGACACGACGACAGAAACATC 3′	60	111
uc004bsl.1	F:5′ AGAGCAGTATGTGGCACCTTT 3′ R:5′ ACCCGCACATCCATGTAGTA 3′	60	178
β-actin(H)	F:5′ GTGGCCGAGGACTTTGATTG 3′ R:5′ CCTGTAACAACGCATCTCATATT 3′	60	73

### Coding–Noncoding Gene Coexpression Network

The coding–noncoding gene coexpression network was constructed based on the correlation analysis between the differentially expressed lncRNAs and mRNAs. The algorithm has been previously described (Liao et al., 2011). The Pearson correlation coefficient between lncRNAs and coding genes was calculated based on the selected lncRNAs and mRNAs expression levels. The parameter is Pearson correlation coefficient (abs) ≥ 0.8, p-value ≤ 0.05, and FDR ≤ 0.05. The coexpression network was illustrated by Cytoscape (v2.8.3) (Institute of Systems Biology, Seattle, US).

### Statistics

Data are presented as mean ± standard error of the mean (SEM) and were analyzed by the statistics software SPSS (SPSS Inc, version 22.0, Chicago, IL, USA). Two-tail Student’s t-test was used to calculate the differences between DCM and ICM group. p-value of <0.05 were regarded as statistically significant. The association between lncRNAs and clinical data was assessed using the Pearson correlation coefficient.

## Results

### Baselines Characteristics

A total of 20 individuals who participated in the study were diagnosed with ICM (n = 9) or DCM (n = 11) at hospital. Baseline characteristics of study population are shown in **Table 2**. In this study, patients were predominantly men (age 59.80 ± 12.81 years) accounting for 90% (18/20). Sixty-five percent patients suffered from hypertension and 25% from diabetes. Patients with DCM showed a lower EF (p < 0.005) and higher left ventricular end diastolic diameter (LVEDD) (p < 0.000) and left ventricular end systolic dimension (LVESD) (p < 0.000) than those with ICM. **Figure S1** presents the representative echocardiograms of DCM and ICM patients. The mean values of pro-Brain Natriuretic Peptide were 4,755.05 ± 5,432.78 ng/l. Most patients received recommended pharmacological therapy for CHF (beta-blockers, 95%; angiotensin-converting enzyme inhibitors or angiotensin receptor blocker, 60%; diuretics, 70%).

**Table 2:** Demographic and clinical characteristics of the study population.

	Overall (N = 20)	ICM (N = 9)	DCM (N = 11)	*p* value
Age (years)	59.80 ± 12.81	62.33 ± 8.26	57.73 ± 15.70	0.439
Male *n* (%)	18 (90.0)	8 (88.9)	10(90.9)	0.881
Hypertension *n* (%)	13 (65)	5 (55.6)	8 (72.7)	0.423
Diabetes *n* (%)	5 (25)	3 (33.3)	2 (18.2)	0.436
NYHA				
II	9 (45.0)	4 (44.4)	5 (45.4)	
III	8 (40.0)	4 (44.4)	4 (36.4)	
IV	3 (15.0)	1 (11.2)	2 (18.2)	
Systolic BP (mmHg)	119.20 ± 19.60	119.00 ± 20.89	119.36 ± 19.51	0.968
Diastolic BP (mmHg)	69.95 ± 9.94	71.78 ± 9.431	68.45 ± 10.55	0.472
Heart rate (per min)	77.50 ± 14.61	79.44 ± 17.01	75.91 ± 12.95	0.604
LVEF (%)	33.10 ± 6.45	37.44 ± 4.64	29.55 ± 5.56	0.003**
LVEDD (mm)	68.60 ± 11.37	59.22 ± 3.31	76.27 ± 9.63	0.000**
LVESD (mm)	58.00 ± 11.63	48.22 ± 3.15	66.00 ± 9.61	0.000**
NT-proBNP (ng/l)	4,755.05 ± 5,432.78	5,505.11 ± 7,707.28	4,141.36 ± 2,763.38	0.590
Medication				
β-Blockers *n* (%)	19(95.0)	8 (88.9)	11 (100.0)	0.257
ACEI or ARB *n* (%)	12(60.0)	4 (44.4)	8 (72.7)	0.199
Diuretics *n* (%)	14(70.0)	6 (66.7)	8 (72.7)	0.769

Values are mean with SD (±) or absolute numbers with relative frequencies (%) at the time of study enrolment. LVEF, left ventricular ejection fraction; LVEDD, left ventricular end diastolic diameter; LEVSD, left ventricular end diastolic diameter pro; ACEI, inhibitors of angiotensin converting enzyme; ARB, angiotensin receptor blocker; BP, blood pressure; NT-proBNP, N-terminal pro-B-type natriuretic peptide; NYHA, New York Heart Association. **p < 0.01.

### lncRNA and mRNA Expression Profiles in DCM and ICM

Arraystar Human lncRNA microarray was used to investigate the lncRNA and mRNA expression changes in plasma of patients with DCM and ICM. We identified 3,222 lncRNAs that were significantly dysregulated in DCM patients vs. ICM patients [fold change (FC) ≥ 2.0, p < 0.05] with 1,637 upregulated and 1,585 downregulated. The top 20 most significant up- and downregulated lncRNAs were shown in **Tables S2** and **S3**.

**Figure 1:**
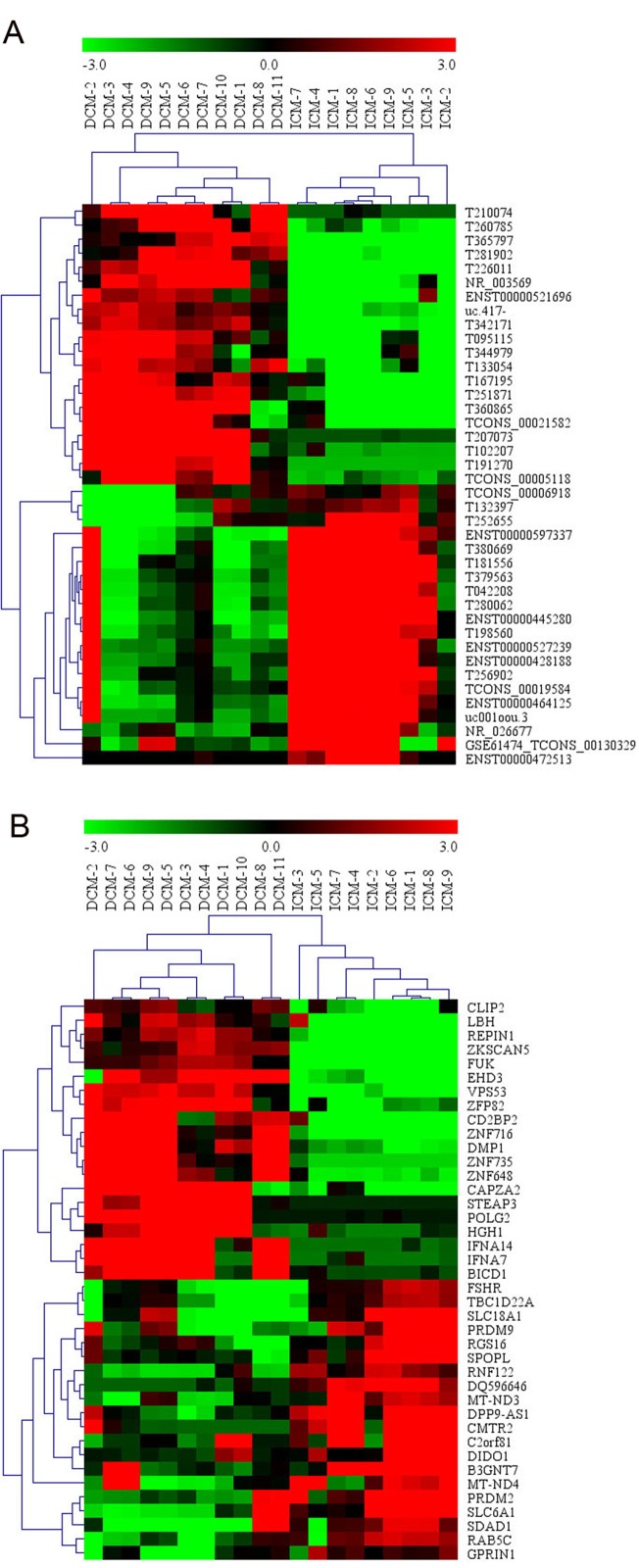
The heat map and hierarchical clustering analysis of long noncoding RNAs (lncRNAs) **(A)** and messenger RNAs (mRNAs) **(B)** that were differentially expressed between the peripheral plasma samples from dilated cardiomyopathy (DCM) and ischemic cardiomyopathy (ICM) patients. Top 20 upregulated and top 20 downregulated results were filtered with fold change (FC) ≥ 2.0 and p 0.05. Expression values are represented in shades of red and green, indicating expression above and below the relative expression respectively. −3.0, 0, and 3.0 are FCs in the corresponding spectrum. The magnitude of deviation from the median is represented by the color saturation.

**Figure 2:**
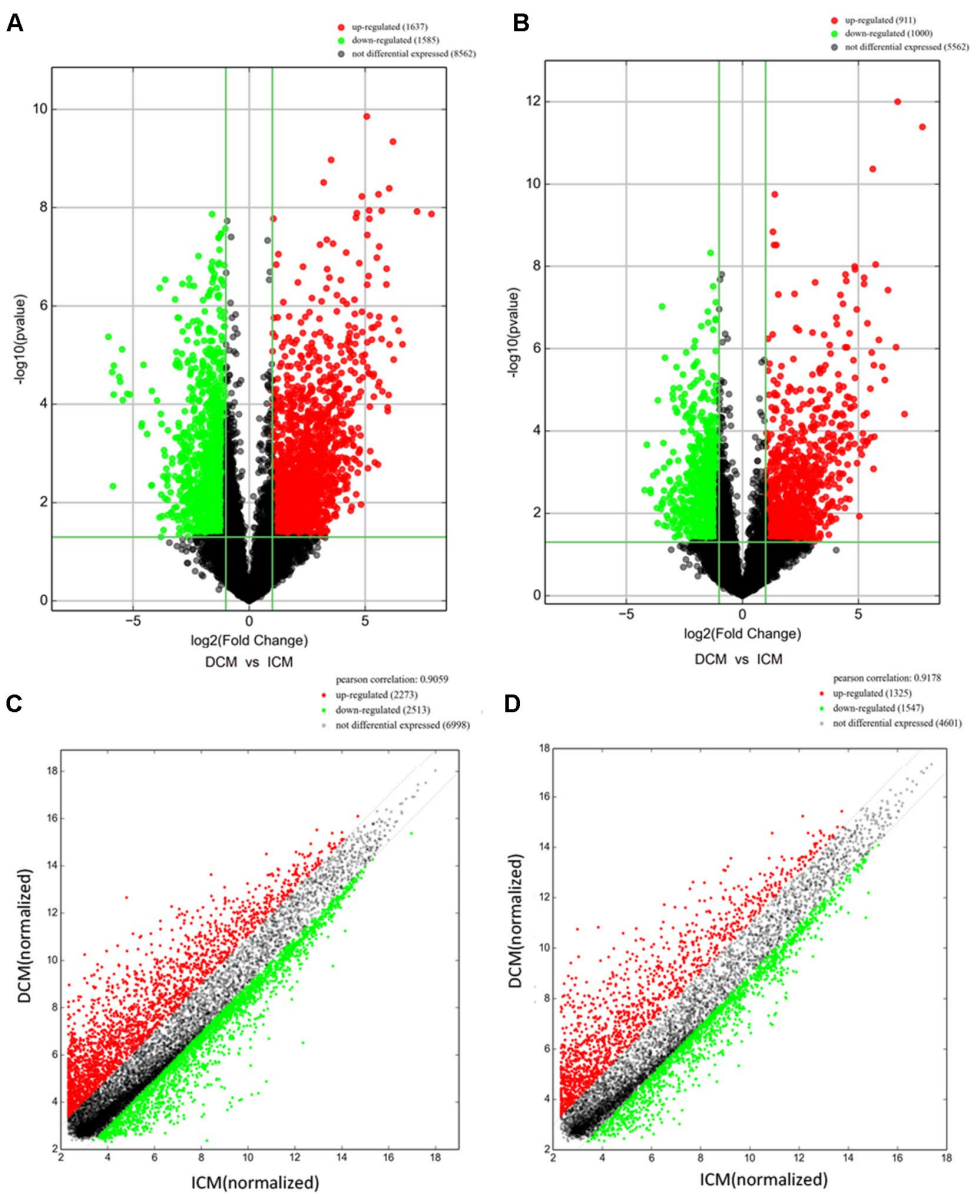
The volcano plots and scatter plots of long noncoding RNA (lncRNA) **(A, C)** and messenger RNA (mRNA) **(B, D)** expression variation between the dilated cardiomyopathy (DCM) and ischemic cardiomyopathy (ICM) patients. Expression values of 11,784 lncRNA and 7,473 mRNA in DCM and ICM patients were converted to log_2_ (fold change) and were compared to −log_10_ (p-value) using a volcano plot. A threshold of p 0.05 and fold change ≥2.0 (The red dots represented upregulated, the green dots represented downregulated). Scatter plots indicated the normalized signal values of the plasma sample (log_2_ scaled).The red dots represented upregulation and fold change ≥ 2.0, the green dots represented downregulation and fold change ≥ 2.0.

Among these lncRNAs transcripts, T342171 was the most upregulated one with an FC of 231.48611, while ENST00000445280 was the most downregulated lncRNA with an FC of 66.66290. The lncRNA and mRNA expression variation between DCM and ICM were shown in a heat map (**Figure 1A**) and the clustering analysis in different groups was presented with the volcano plots and the scatter plots (**Figures 2A, C**).

Additionally, we identified 1,911 significantly differentially expressed mRNAs in which 911 were upregulated, while 1,000 were downregulated (FC ≥ 2.0, p < 0.05) in DCM patients compared with ICM patients. The top 20 significant up- and downregulated mRNAs are showed in **Tables S4** and **S5**. The most upregulated mRNA transcript was FUK (NM_145059), with an FC of 216.16425 while the most downregulated was SLC6A1 (NM_003042), with an FC of 18.49104. The heat map was shown in **Figure 1B**. The variation of mRNA transcripts expression between DCM and ICM group was presented with the volcano plots and the scatter plots (**Figures 2B, D**).

### Chromosome Distribution of Differentially Expressed lncRNAs in DCM and ICM Group

**Figure 3:**
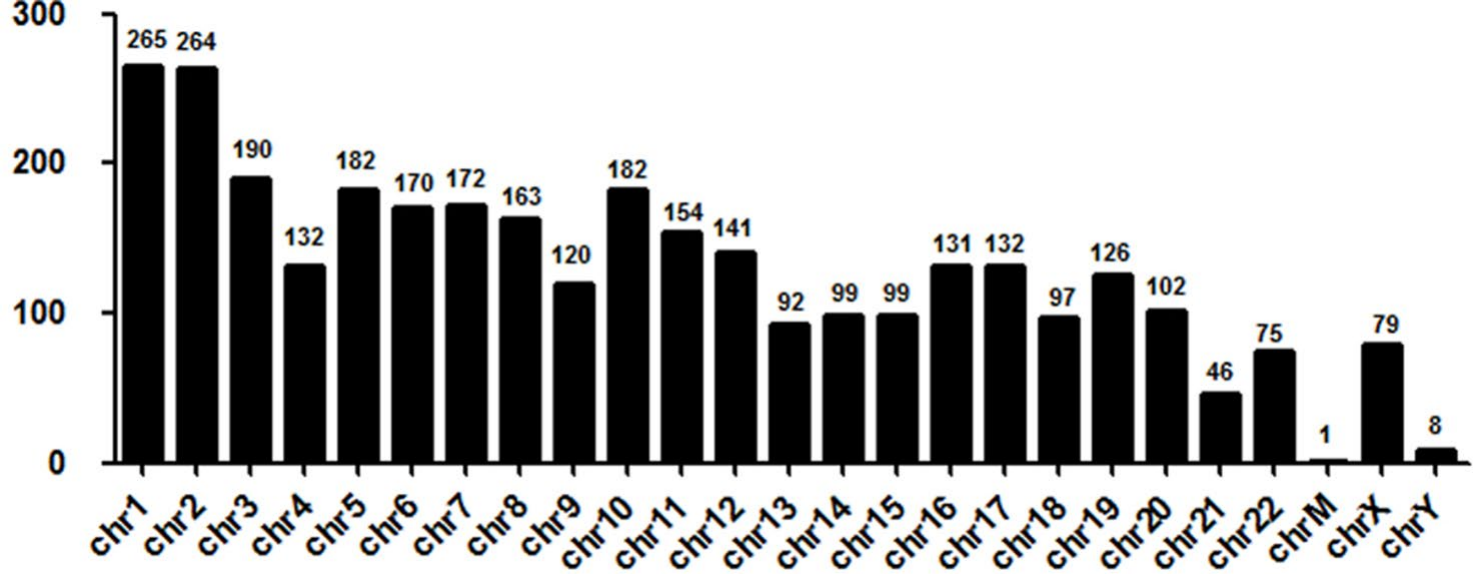
Chromosome distribution of long noncoding RNAs (lncRNAs) differentially expressed in plasma between the DCM and ICM patients. chr, chromosome; M, mitochondrial.

Next, we evaluated the chromosome distribution of lncRNAs differentially expressed in plasma between patients with DCM and ICM. As shown by **Figure 3**, large amount of differentially expressed lncRNAs were located in chromosome 1 (265 lncRNA) and 2 (264 lncRNA) while only one lncRNA named uc022bqs.1 is in mitochondrial genome.

### Gene Ontology and KEGG Pathway Enrichment Analysis

**Figure 4:**
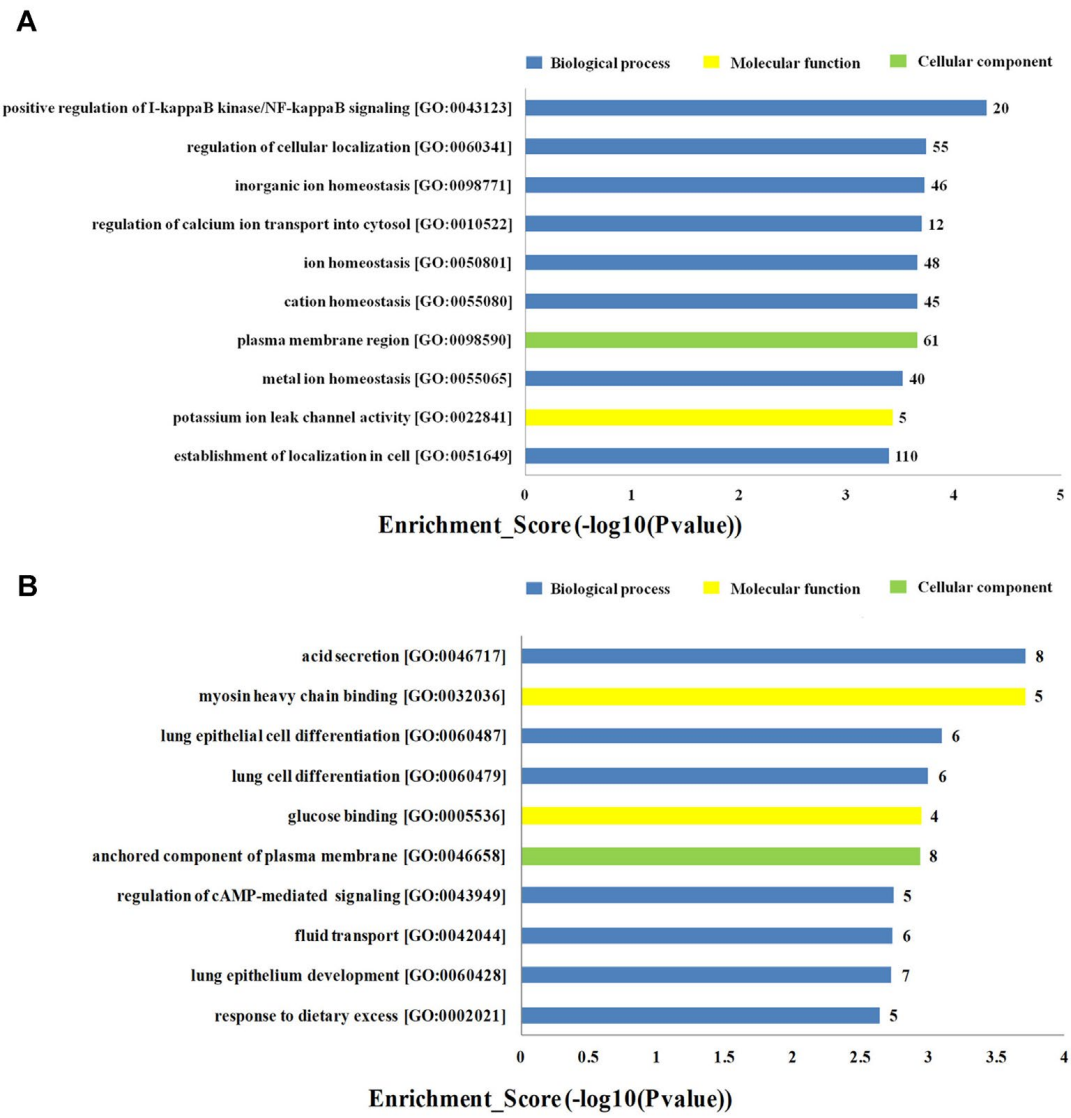
Top 20 GO terms for the differences in coexpressed long noncoding RNA (lncRNA) genes in the dilated cardiomyopathy (DCM) and ischemic cardiomyopathy (ICM) patients. The top 10 Gene Ontology (GO) terms that upregulated genes correlated with **(A)**. The top 10 GO terms that downregulated genes correlated with **(B)**. The bar plot shows the top 10 enrichment score [−log10(p-value)] value of the significant enrichment GO terms.

To investigate the biological functions of lncRNAs in plasma of HF patients with distinct etiology, we performed a functional enrichment analysis of the mRNAs coexpressed with each of the differentially expressed lncRNAs. The enriched functional terms were used as the predicted terms for each given lncRNA. **Figures 4A, B** showed the top 10 upregulated and downregulated GO terms respectively for the differences in mRNAs coexpressed with lncRNAs in DCM and ICM patients. The most enriched upregulated functional terms included positive regulation of I-kappaB kinase/nuclear factor (NF)-kappaB signaling (GO:0043123), regulation of cellular localization (GO:0060341), and inorganic ion homeostasis (GO:0098771)(**Figure 4A**). **Figure 4B** showed the top 10 downregulated genes GO analysis which consist of acid secretion (GO:0046717) and myosin heavy chain binding (GO:0032036).

**Figure 5:**
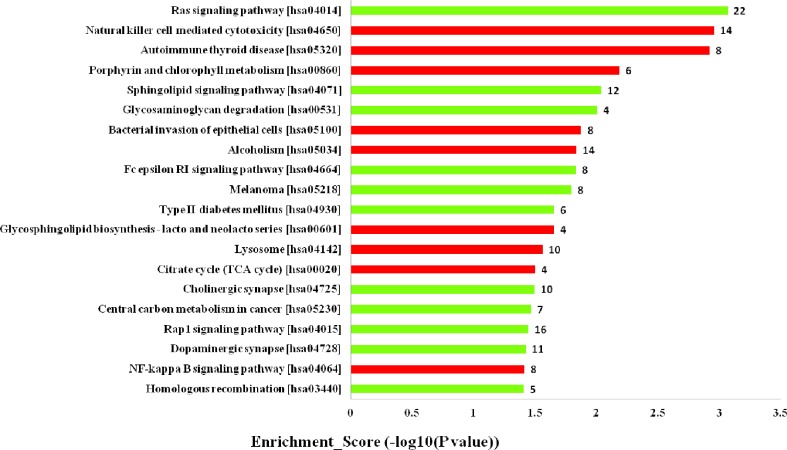
Kyoto Encyclopedia of Genes and Genomes (KEGG) pathways analysis. Top 20 pathways for the differences in long noncoding RNA (lncRNA) genes coexpressed in the dilated cardiomyopathy (DCM) and ischemic cardiomyopathy (ICM) patients. The red bars are associated with upregulated pathways, the green bars are associated with downregulated pathways. The bar plot shows the top 20 enrichment score [−log10(p-value)] value of the significant enrichment gene ontology terms.

Furthermore, the KEGG pathway analysis revealed that the upregulated mRNAs coexpressed with lncRNAs were mainly enriched in natural killer cell mediated cytotoxicity, autoimmune thyroid disease and porphyrin and chlorophyll metabolism. While the downregulated terms were primarily involved in the regulation of ras signaling pathway, sphingolipid signaling pathway and glycosaminoglycan degradation (**Figure 5**). The top 20 KEGG pathways are listed in **Figure 5**.

### Validation of the Microarray Data by Quantitative Real-Time PCR

**Figure 6:**
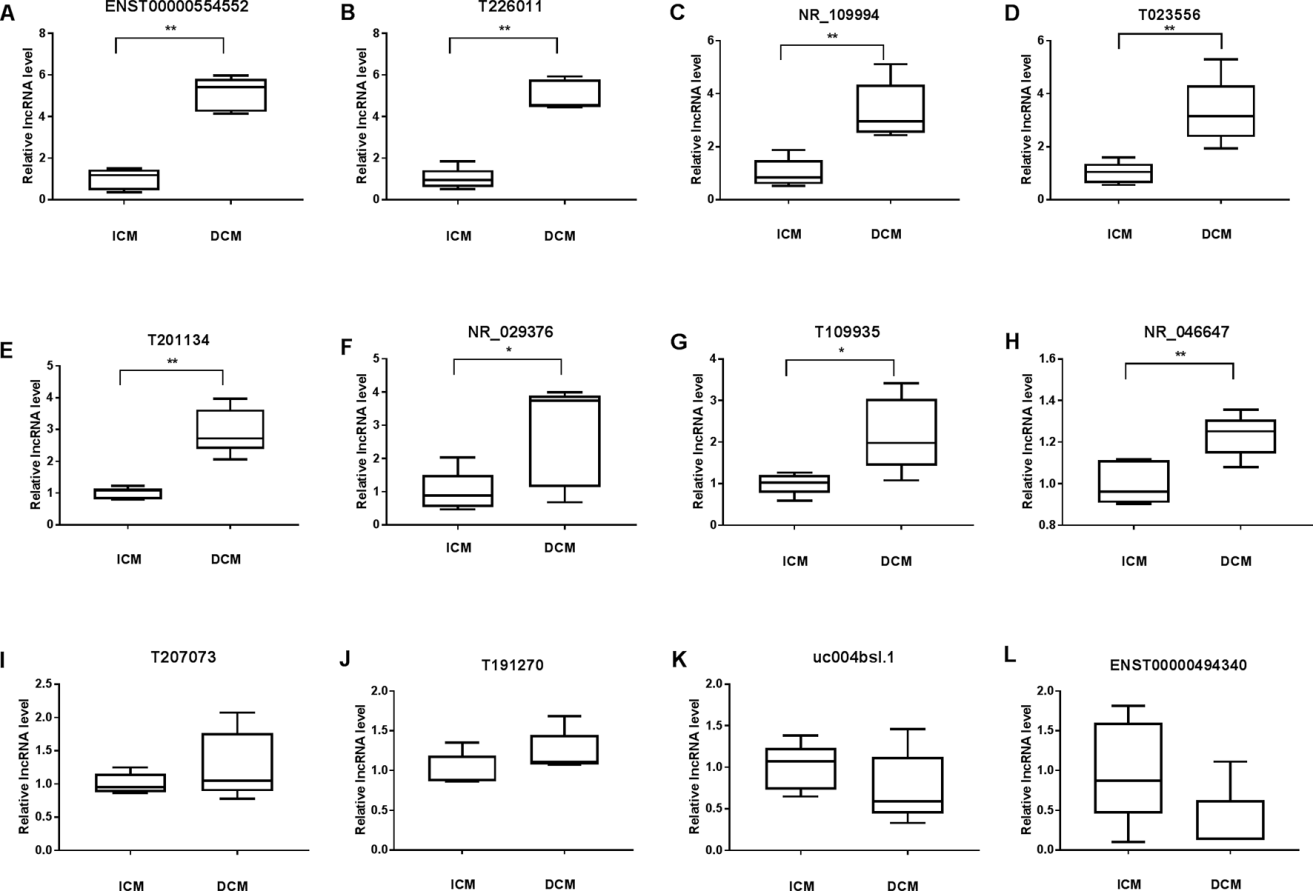
Validation of the differential expression of long noncoding RNAs (lncRNAs) by quantitative real-time PCR (qRT-PCR) (A–L). Levels of the lncRNA (T226011, ENST00000554552, T201134, T023556, T109935, NR_109994, NR_046647, NR_029376, ENST00000494340, T191270, T207073, uc004bsl.1) in plasma of patients with dilated cardiomyopathy (DCM) or ischemic cardiomyopathy (ICM). DCM group, n = 11; ICM group, n = 9. *p 0.05 versus control, **p 0.01 versus control.

To validate the lncRNAs expression alterations detected by microarray, a total of 12 differentially expressed lncRNAs (T226011, ENST00000554552, T201134, T023556, T109935, NR_109994, NR_046647, NR_029376, ENST00000494340, T191270, T207073, uc004bsl.1) were selected and their expressions examined by RT-PCR. Consistent with the microarray chip data, there were more than five fold increase of ENST00000554552 and T226011 in DCM group compared with the ICM group (**Figures 6A, B**). Meanwhile, circulating NR_109994, T023556, T201134, NR_029376, T109935, and NR_046647 increased 3.34-fold, 3.34-fold, 2.95-fold, 2.76-fold, 2.19-fold, and 1.23-fold respectively in DCM group compared with ICM (**Figures 6C–H**). Other four lncRNAs showed no significant differences between the two groups (**Figures 6I–L**).

### lncRNA–mRNA Network Analysis

**Figure 7:**
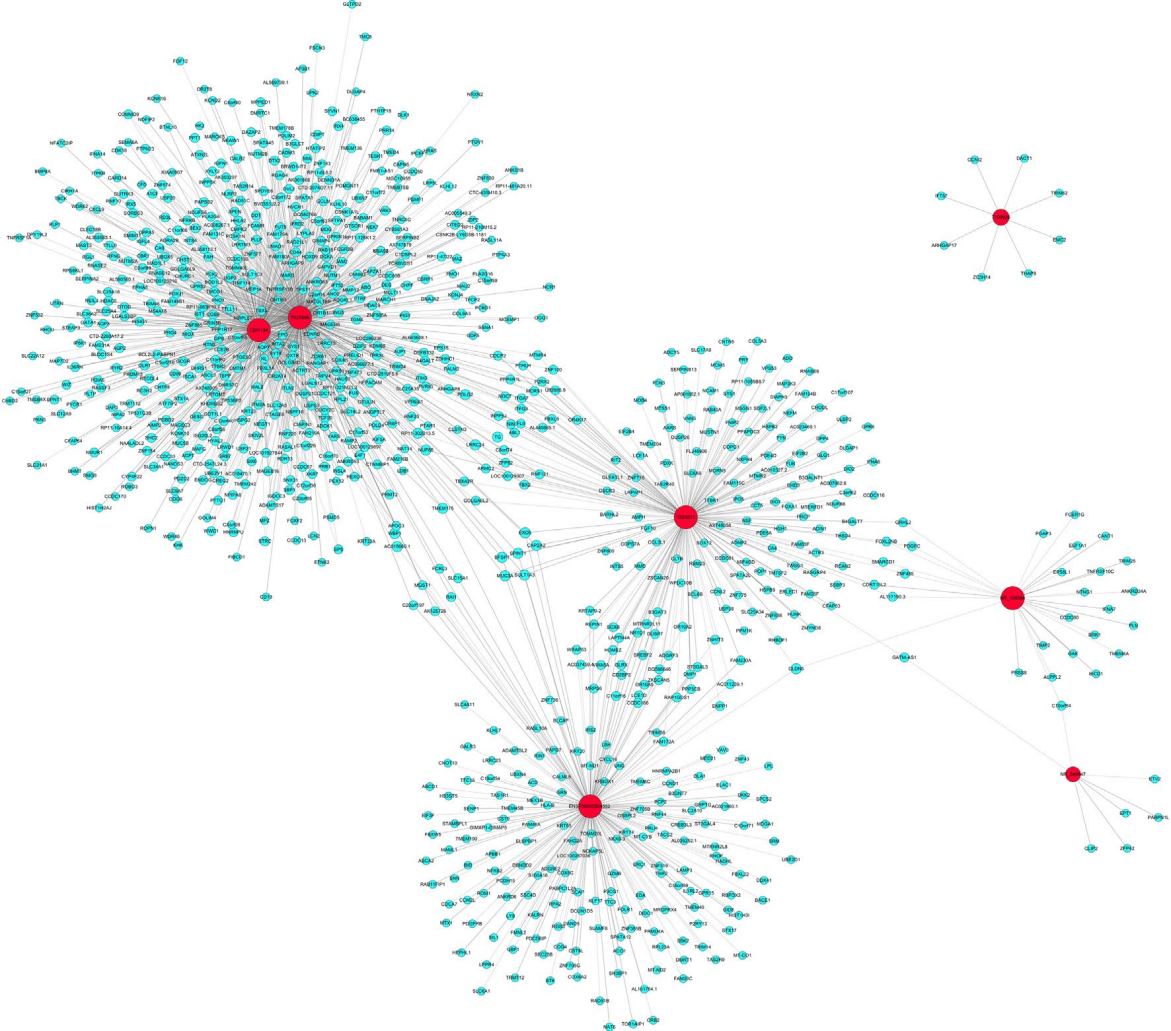
Coding–noncoding gene coexpression networks network of the seven validated long noncoding RNAs (lncRNAs) and their correlated messenger RNAs (mRNAs). The network represents coexpression correlations between seven lncRNAs and their correlated mRNAs. Red nodes represent lncRNAs. Blue nodes represent correlated mRNAs. The real lines between lncRNAs and mRNAs mean positive correlation, while the dotted lines mean negative correlation.

To further elucidate the relevant functions of the target genes, a functional network was constructed based on the GO analysis using Cytoscape. 7 validated differentially expressed lncRNA in DCM group compared with ICM group were used to construct a coding–noncoding gene coexpression network. **Figure 7** showed the network profiles based on these 7 lncRNAs and 906 mRNAs. Our results showed that one lncRNA may correlate with 7–517 mRNAs. lncRNA T023556 is correlated with 517 mRNAs in which 227 upregulated and 290 downregulated. lncRNA T201134 is correlated with 401 mRNAs with 161 upregulated and 240 downregulated. Total of 203 mRNAs, including 103 upregulated and 100 downregulated mRNAs are predicted to correlate with lncRNA T226011. As shown by **Figure 7**, lncRNA T109935 and NR_046647 were correlated with eight and seven mRNAs respectively.

### Associations Between Upregulated lncRNAs and the Clinical Index

The relationship between the rising lncRNAs and the clinical index were also studied using Pearson linear correlation analysis. We choose three upregulated lncRNAs including ENST00000554552,T226011 and NR_109994. Pearson linear correlation coefficient showed that there were no significant correlations between EF, pro-Brain Natriuretic Peptide, and these lncRNAs (p > 0.05). There were moderate positive correlations between LVEDD, LVESD, and lncRNAs (0.4 < r < 0.6, p < 0.05) (**Table 3**).

**Table 3:** Correlations between three long noncoding RNAs (lncRNAs) and the clinical data including EF, LVEDD, LVESD, and NT-proBNP by Pearson linear correlation analysis.

	EF		LVEDD		LVESD		NT-proBNP	
	*r*	*p* value	*r*	*p* value	*r*	*p* value	*r*	*p* value
ENST00000554552	−0.210	0.203	0.487	0.003**	0.489	0.003**	0.076	0.649
T226011	−0.286	0.084	0.593	0.000**	0.575	0.000**	−0.018	0.916
NR_109994	−0.200	0.227	0.550	0.001**	0.468	0.004**	0.111	0.506

**p < 0.01.

## Discussion

The present findings revealed for the first time the specific expression pattern of both protein-coding and lncRNAs in plasma of HF patients due to DCM and ICM, confirming that new biomarkers could be reliably identified using microarray-based approaches. As previously reported, transcriptional profiling has been utilized extensively in human HF to explore the new pathways involved in the complex disease (Hannenhalli et al., 2006), to identify novel biomarkers for better diagnostic and prognostic accuracy (Kittleson et al., 2004), and to evaluate the treatment responses of different medications or implanted devices (Margulies et al., 2005; Matkovich et al., 2009; Ramani et al., 2011). Results from animal diseases models demonstrated the vital roles of lncRNAs in cardiac hypertrophy (Han et al., 2014; Wang et al., 2016), MI (Ishii et al., 2006; Qu et al., 2017), coronary artery disease (Gao et al., 2015), vascular injury (Kim et al., 1994), atherosclerotic plaques (Han et al., 1996), and high blood pressure (Tragante et al., 2014). Recently, RNAseq was used to determine the lncRNAs expression in human hearts (Yang et al., 2014; Saddic et al., 2017; Schiano et al., 2017). The lncRNA expression landscape could distinguish cardiomyopathic samples before and after treatment (Yang et al., 2014; Saddic et al., 2017) and specific lncRNA profile was found in hearts from HF patients with DCM and restrictive cardiomyopathy (Schiano et al., 2017). Recent microarray analysis demonstrated the lncRNA dysregulation in hearts suffered from ischemic HF compared to control hearts (Greco et al., 2016), as well as in hearts with DCM from control (Li et al., 2018). Therefore, lncRNAs are closely involved in the cardiac pathophysiological processes. We presented the specific expression pattern of both mRNAs and lncRNAs in plasma of HF patients subjected to DCM and ICM, supporting the notion that new lncRNAs biomarkers in plasma may be applied to discriminate the etiology of HF from DCM and ICM.

Further results showed positive relationships in lncRNA expressions between tissues and plasmas in patients with hepatocellular carcinoma (Wang et al., 2018) and colorectal cancer (Neve et al., 2018). Compared to the tissues, isolation of nucleic acids from plasma serves as an ideal method for early and differentiation diagnosis, evaluation the medication responses, and assessment of the prognosis. It is reported that specific lncRNAs, aberrantly expressed in the plasma of pregnant women with typical fetal congenital heart defects, may be used as novel biomarkers for prenatal diagnosis of fetal CHD (Gu et al., 2016). Circulating lncRNAs could also be used as biomarkers of LV diastolic function and remodeling in patients with well-controlled type 2 diabetes (De Gonzalo-Calvo et al., 2016) or to predicts survival in patients with HF (Kumarswamy et al., 2014). Therefore, lncRNA expression in the plasma may serve as convenient and novel diagnostic markers in heart diseases. The present study showed a signature lncRNA profile in plasma of patients with DCM and ICM. In addition, several differentially expressed lncRNAs were chosen for qRT-PCR validation, and the results were consistent with the microarray analysis findings. The moderate positive correlations between LVEDD, LVESD and some upregulated lncRNAs also suggested the associations between lncRNAs and chronic cardiac enlargement. Recently, Zhang et al. investigated the specific circulating lncRNAs in patients with DCM compared to that of the control (Zhang et al., 2019). They found both levels of circulating lncRNA ENST00000507296 and ENST00000532365 were significantly correlated with the cardiac function. In this study, circulating lncRNA ENST00000507296 level was also upregulated in DCM group compared to ICM group, suggesting the role of this lncRNA in DCM compared to ICM. How these molecules are involved in the onset or progress of cardiac dysfunction are still elusive. Due to the variability in etiologies, presentations of diseases and treatment strategies, convenient markers for differential diagnosis is of great importance in clinical settings. Our data indicated that different panel or expression signature of lncRNA may serve as a good diagnostic marker for making a distinction of HF patients due to DCM from ICM.

Previous report (Kumarswamy et al., 2014) showed that most of the lncRNAs that originate from mitochondrial genome were downregulated in plasma of patients with high LV remodeling 12 months after MI and the strongest association were found between downregulation of uc022bqs.1 and severe LV remodeling. We found that uc022bqs.1 expression is decreased in DCM compared to ICM. Therefore, downregulation of uc022bqs.1 may partly explain the much higher LVEDD and LVESD in DCM than ICM, indicating a more sever remodeling in DCM than the ICM. We also found large amount of differentially expressed lncRNAs in other chromosomes especially chromosome1 and 2, how are these lncRNAs involved in the progress of HF with different etiologies need further exploration.

lncRNAs were reported to play crucial roles in regulating gene expression, transcription, posttranscription, and epigenetic modification (Chen, 2016). Our GO and KEGG pathway analyses predicted the potential functions of the differentially expressed lncRNAs identified in this study. GO analysis revealed that these lncRNAs are profoundly involved in multiple biological processes including upregulated functional terms associated with acid secretion, lung epithelial cell differentiation and lung cells differentiation, and downregulated positive regulation of I-kappaB kinase/NF-kappa B signaling, regulation of cellular localization, and inorganic ion homeostasis. Compelling evidences demonstrated the role of NF-kappa B signaling in the regulation of immune cell maturation and inflammation (Baldwin, 2012; Hoesel and Schmid, 2013; Gerondakis et al., 2014), and acute hypoxia and reperfusion injury (Regula et al., 2004; Zhou et al., 2008). Prolonged activation of NF-kappa B was proved to promote HF by triggering chronic inflammation signals and enhanced endoplasmic reticulum stress responses and cell death (Purcell et al., 2001; Kawamura et al., 2005; Kawano et al., 2006; Gordon et al., 2011). Many reports confirmed the critical role of inflammation in ventricular remodeling and final HF post MI (Westman et al., 2016). The present results suggested that inflammation triggered by NF-kappa B signaling may dominate in ICM other than DCM. In addition, increased myosin heavy chain binding and ATP utilization lead to the hypercontractile sarcomere in hypertrophic cardiomyopathy (Reiser et al., 2001; Rajabi et al., 2007). In our molecular function analysis results, myosin heavy chain binding was upregulated in DCM group compared to ICM, suggesting more profound involvement of lncRNA molecules associated with this pathway in DCM from ICM.

The KEGG pathway analysis revealed that the upregulated mRNAs coexpressed with lncRNAs were mainly enriched in natural killer cell mediated cytotoxicity, while the downregulated terms in ras signaling pathway and sphingolipid signaling pathway. Among these, natural killer cell mediated cytotoxicity was reported to decrease in advanced HF patients (Vredevoe et al., 1995; Doering et al., 1997). The present result showed mRNAs coexpressed with lncRNAs associated with natural killer cell mediated cytotoxicity downregulated in ICM plasma compared to DCM group. Sphingolipids was reported to contribute to pathophysiological mechanisms by modifying signaling and metabolic pathways (Rodriguez-Cuenca et al., 2017). Recent findings demonstrated its involvement in cardiovascular diseases (Fenger et al., 2011; Sasset et al., 2016), and diabetes (Jessup et al., 2011). Data from animal experiment underlined a cardioprotective function of S1P signaling in ischemia reperfusion injury (Theilmeier et al., 2006). Our results further showed that the expression of mRNAs coexpressed with lncRNAs associated with sphingolipid signaling pathway in plasma may differ HF patients with DCM from ICM. Further studies are needed to explore how other dysregulated mRNAs coexpressed with lncRNAs enriched signaling pathways contribute to the progression of HF.

Many studies demonstrated that lncRNAs act as a potentially new and crucial regulators in gene expression, transcription, posttranscription, and epigenetics levels (Chen, 2016). Among these, modulation of mRNA represents one of the most important actions of lncRNA in diseases. Our coexpression network analysis of the seven validated lncRNA and their correlated mRNAs showed that lncRNAs may regulate several to hundreds of mRNAs. Recent study revealed that some lncRNAs with the central topological features were found in the HF-related lncRNA–mRNA network (Fan et al., 2018). The coexpression network indicates that the interregulation of lncRNAs and mRNAs is involved in the pathophysiology of HF and warrants further investigations.

In conclusion, the present study revealed a specific expression pattern of both protein-coding and lncRNAs in plasma of HF patients with DCM and ICM. Moreover, the differentially expressed lncRNAs are involved in several specific biological processes, molecular functions and cellular components and regulate some signaling pathways which may uncover the different mechanisms in which HF is attributed. Therefore, these findings may lead to new insights into the novel diagnostic and therapeutic targets for HF patients with DCM and ICM.

## Data Availability Statement

The datasets in generated for this study can be found in the Gene expression Omnibis, accession GSE138678.

## Ethics Statement

The study conforms to the ethical guidelines of the 1975 Declaration of Helsinki. The Ethics Committee of Shanghai East Hospital, Tongji University School of Medicine approved the protocol of this study and all enrolled patients gave the written informed consent.

Written informed consent was obtained for each participants according to the ethical guidelines of the 1975 Declaration of Helsinki. These consent are available from the corresponding author on reasonable request.

## Author Contributions

XZ and HF conceived and designed the experiments. ZL conceived the experiments. XZ and FL wrote the paper. FL, XG, PY, AY, and LH performed the experiments. HC and TC provided help in imaging. QM, PY, LZ, and XL analyzed and interpreted the data. QM, JC, YL, and HH contributed to collection of the materials. All authors read and approved the final manuscript.

## Funding

This work was supported by National Nature Science Foundation of China (81670458, 81470393, and 81370434), The National Key Research and Development Program of China (2017YFA0105600), Key Discipline of the Health Industry Project of Pudong Health Bureau of Shanghai (PWZxk2017-01), and the Science and Technology Commission of Shanghai Municipality (17431906600) Shanghai Three-year plan on promoting TCM development (ZY (2018-2020)-FWTX-2007), The key clinical disciplines construction of Shanghai (2017zz02017).

## Conflict of Interest

The authors declare that the research was conducted in the absence of any commercial or financial relationships that could be construed as a potential conflict of interest.

## References

[B1] Armstrong P. W.WEST Steering Committee (2006). A comparison of pharmacologic therapy with/without timely coronary intervention vs. primary percutaneous intervention early after ST-elevation myocardial infarction: the WEST (Which Early ST-elevation myocardial infarction Therapy) study. Eur. Heart J. 27, 1530–1538. 10.1093/eurheartj/ehl088 16757491

[B2] Baldwin A. S. (2012). Regulation of cell death and autophagy by IKK and NF-κB: critical mechanisms in immune function and cancer. Immunol. Rev. 246, 327–345. 10.1111/j.1600-065X.2012.01095.x 22435564

[B3] Chen L. L. (2016). Linking long noncoding RNA localization and function. Trends Biochem. Sci. 41, 761–772. 10.1016/j.tibs.2016.07.003 27499234

[B4] Dangwal S. Schimmel K. Foinquinos A. Xiao K. Thum T. (2016). Noncoding RNAs in heart failure. Handb. Exp. Pharmacol. 243, 423–445. 10.1007/164_2016_99 27995387

[B5] De Gonzalo-Calvo D. Kenneweg F. Bang C. Toro R. van der Meer R. W. Rijzewijk L. J. (2016). Circulating long-non coding RNAs as biomarkers of left ventricular diastolic function and remodelling in patients with well-controlled type 2 diabetes. Sci. Rep. 6, 37354. 10.1038/srep37354 27874027 PMC5118808

[B6] Di Salvo T. G. (2015). Epigenetic regulation in heart failure: part I RNA. Cardiol. Rev. 23, 213–228. 10.1097/CRD.0000000000000071 25839993

[B7] Doering L. V. Vredevoe D. L. Woo M. A. Fonarow G. C. Skotsko C. Bonavida B. (1997). Predictors of natural killer cell-mediated cytotoxicity deficiency in advanced heart failure secondary to either ischemic or idiopathic dilated cardiomyopathy. Am. J. Cardiol. 80, 234–236. 10.1016/s0002-9149(97)00332-9 9230174

[B8] Fan Z. Gao S. Chen Y. Xu B. Yu C. Yue M. (2018). Integrative analysis of competing endogenous RNA networks reveals the functional lncRNAs in heart failure. J. Cell. Mol. Med. 22, 4818–4829. 10.1111/jcmm.13739 30019841 PMC6156393

[B9] Felker G. M. Thompson R. E. Hare J. M. Hruban R. H. Clemetson D. E. Howard D. L. (2000). Underlying causes and long-term survival in patients with initially unexplained cardiomyopathy. N. Engl. J. Med. 342, 1077–1084. 10.1056/NEJM200004133421502 10760308

[B10] Fenger M. Linneberg A. Jørgensen T. Madsbad S. Søbye K. Eugen-Olsen J. (2011). Genetics of the ceramide/sphingosine-1-phosphate rheostat in blood pressure regulation and hypertension. BMC Genet. 12, 44. 10.1186/1471-2156-12-44 21569466 PMC3115901

[B11] Follath F. Cleland J. G. Klein W. Murphy R. (1998). Etiology and response to drug treatment in heart failure. J. Am. Coll. Cardiol. 32, 1167–1172. 10.1016/s0735-1097(98)00400-8 9809921

[B12] Gao W. Zhu M. Wang H. Zhao S. Zhao D. Yang Y. (2015). Association of polymorphisms in long non-coding RNA H19 with coronary artery disease risk in a Chinese population. Mutat.Res. 772, 15–22. 10.1016/j.mrfmmm.2014.12.009 25772106

[B13] Gerondakis S. Fulford T. S. Messina N. L. Grumont R. J. (2014). NF-κB control of T cell development. Nat. Immunol. 15, 15–25. 10.1038/ni.2785 24352326

[B14] Gordon J. W. Shaw J. A. Kirshenbaum L. A. (2011). Multiple facets of NF-κB in the heart: to be or not to NF-κB. Circ. Res. 108, 1122–1132. 10.1161/CIRCRESAHA.110.226928 21527742

[B15] Greco S. Zaccagnini G. Perfetti A. Fuschi P. Valaperta R. Menicanti L. (2016). Long noncoding RNA dysregulation in ischemic heart failure. J. Transl. Med. 14, 183. 10.1186/s12967-016-0926-5 27317124 PMC4912721

[B16] Gu M. Zheng A. Tu W. Zhao J. Zhu J. Pan Y. (2016). Circulating LncRNAs as novel, non-invasive biomarkers for prenatal detection of fetal congenital heart defects. Cell. Physiol. Biochem. 38, 1459–1471. 10.1159/000443088 27035723

[B17] Han D. K. Khaing Z. Z. Pollock R. A. Haudenschild C. C. Liau G. (1996). H19, a marker of developmental transition, is reexpressed in human atherosclerotic plaques and is regulated by the insulin family of growth factors in cultured rabbit smooth muscle cells. J. Clin. Invest. 97, 1276–1285. 10.1172/JCI118543 8636440 PMC507181

[B18] Hannenhalli S. Putt M. E. Gilmore J. M. Wang J. Margulies K. B. Cappola T. P. (2006). Transcriptional genomics associates FOX transcription factors with human heart failure. Circulation 114, 1269–1276. 10.1161/CIRCULATIONAHA.106.632430 16952980

[B19] Han P. Li W. Lin C. H. Yang J. Lin C. Y. Lin C. J. (2014). A long noncoding RNA protects the heart from pathological hypertrophy. Nature 514, 102–106. 10.1038/nature13596 25119045 PMC4184960

[B20] Hoesel B. Schmid J. A. (2013). The complexity of NF-κB signaling in inflammation and cancer. Mol. Cancer 12, 86. 10.1186/1476-4598-12-86 23915189 PMC3750319

[B21] Ishii N. Ozaki K. Sato H. Mizuno H. Hori M. Saito S. (2006). Identification of a novel non-coding RNA, MIAT, that confers risk of myocardial infarction. J. Hum. Genet. 51, 1087–1099. 10.1007/s10038-006-0070-9 17066261

[B22] Jessup C. F. Bonder C. S. Pitson S. M. Coates P. T. (2011). The sphingolipid rheostat: a potential target for improving pancreatic islet survival and function. Endocr. Metab. Immune Disord. Drug Targets 11, 262–272. 10.2174/187153011797881201 21696364

[B23] Kawamura N. Kubota T. Kawano S. Takeshita A. Sunagawa K. (2005). Blockade of NF-kappaB improves cardiac function and survival without affecting inflammation in TNF-alpha-induced cardiomyopathy. Cardiovasc. Res. 66, 520–529. 10.1016/j.cardiores.2005.02.007 15914117

[B24] Kawano S. Kubota T. Monden Y. Tsutsumi T. Tsutsui H. Sunagawa K. (2006). Blockade of NF-kappaB improves cardiac function and survival after myocardial infarction. Am. J. Physiol. Heart Circ. Physio. 291, 1337–1344. 10.1152/ajpheart.01175.2005 16632551

[B25] Kim D. K. Zhang L. Dzau V. J. Pratt R. E. (1994). H19, a developmentally regulated gene, is reexpressed in rat vascular smooth muscle cells after injury. J. Clin. Invest. 93, 355–360. 10.1172/JCI116967 8282806 PMC293782

[B26] Kittleson M. M. Ye S. Q. Irizarry R. A. Minhas K. M. Garcia J. G. Hare J. M. (2004). Identification of a gene expression profile that differentiates between ischemic and nonischemic cardiomyopathy. Circulation. 110, 3444–3451. 10.1161/01.CIR.0000148178.19465.11 15557369

[B27] Kumarswamy R. Bauters C. Lemesle G. de Groote P. Pinet F. Thum T. (2014). Circulating long noncoding RNA, LIPCAR, predicts survival in patients with heart failure. Circ. Res. 114, 1569–1575. 10.1161/CIRCRESAHA.114.303915 24663402

[B28] Kumarswamy R. Thum T. (2013). Non-coding RNAs in cardiac remodeling and heart failure. Circ. Res. 113, 676–689. 10.1161/CIRCRESAHA.113.300226 23989712

[B29] Liao Q. Liu C. Yuan X. Wu Z. Zhao Y. (2011). Large-scale prediction of long non-coding RNA functions in a coding-non-coding gene co-expression network. Nucleic Acids Res. 39, 3864–3878. 10.1093/nar/gkq1348 21247874 PMC3089475

[B30] Li H. Chen C. Fan J. Yin Z. Ni L. Cianflone K. (2018). Identification of cardiac long non-coding RNA profile in human dilated cardiomyopathy. Cardiovasc. Res. 114, 747–758. 10.1093/cvr/cvy012 29365080

[B31] Livak K. J. Schmittgen T. D. (2001). Analysis of relative gene expression data using real-time quantitative PCR and the 2 (-Delta Delta C(T)) method. Methods. 25, 402–408. 10.1006/meth.2001.1262 11846609

[B32] Margulies K. B. Matiwala S. Cornejo C. Olsen H. Craven W. A. Bednarik D. (2005). Mixed messages:transcription patterns in failing and recovering human myocardium. Circ. Res. 96, 592–599. 10.1161/01.RES.0000159390.03503.c3 15718504

[B33] Matkovich S. J. Van Booven D. J. Youker K. A. Margulies K. B. Dorn G. W. (2009). Reciprocal regulation of myocardial microRNAs and messenger RNA in human cardiomyopathy and reversal of the microRNA signature by biomechanical support. Circulation. 119, 1263–1271. 10.1161/CIRCULATIONAHA.108.813576 19237659 PMC2749457

[B34] Matouk I. J. Halle D. Gilon M. Hochberg A. (2015). The non-coding RNAs of the H19-IGF2 imprinted loci: a focus on biological roles and therapeutic potential in lung cancer. J. Transl. Med. 13, 113. 10.1186/s12967-015-0467-3 25884481 PMC4397711

[B35] Neve B. Jonckheere N. Vincent A. Van Seuningen I. (2018). Epigenetic regulation by lncRNAs: an overview focused on UCA1 in colorectal cancer. Cancers (Basel). 10 (11), 440–473. 10.3390/cancers10110440 PMC6266399 30441811

[B36] Peng J. Wu Y. Tian X. Chen L. Jiang Y. (2017). High-throughput sequencing and co-expression network analysis of lncRNAs and mRNAs in early brain injury following experimental subarachnoid haemorrhage. Sci. Rep. 7, 46577. 10.1038/srep46577 28417961 PMC5394545

[B37] Purcell N. H. Tang G. Yu C. Mercurio F. DiDonato J. A. Lin A. (2001). Activation of NF-kappa B is required for hypertrophic growth of primary rat neonatal ventricular cardiomyocytes. Proc. Natl. Acad. Sci. U. S. A. 98, 6668–6673. 10.1073/pnas.111155798 11381115 PMC34410

[B38] Qu X. Du Y. Shu Y. Sun F. Wang Z. Yang B. (2017). MIAT is a pro-fibrotic long non-coding RNA governing cardiac fibrosis in post-infarct myocardium. Sci. Rep. 7, 42657. 10.1038/srep42657 28198439 PMC5309829

[B39] Rajabi M. Kassiotis C. Razeghi P. Taegtmeyer H. (2007). Return to the fetal gene program protects the stressed heart:a strong hypothesis. Heart Fail. Rev. 12, 331–343. 10.1007/s10741-007-9034-1 17516164

[B40] Ramani R. Vela D. Segura A. McNamara D. Taegtmeyer H. McTiernan C. F. (2011). A micro-ribonucleic acid signature associated with recovery from assist device support in 2 groups of patients with severe heart failure. J. Am. Coll. Cardiol. 58, 2270–2278. 10.1016/j.jacc.2011.08.041 22093502 PMC3226759

[B41] Regula K. M. Baetz D. Kirshenbaum L. A. (2004). Nuclear factor-kappaB represses hypoxia-induced mitochondrial defects and cell death of ventricular myocytes. Circulation. 10, 3795–3802. 10.1161/01.CIR.0000150537.59754.55 15596562

[B42] Reiser P. J. Portman M. A. Ning X. H. Schomisch Moravec C. (2001). Human cardiac myosin heavy chain isoforms in fetal and failing adult atria and ventricles. Am. J. Physiol. Heart Circ. Physiol. 280, 1814–1820. 10.1152/ajpheart.2001.280.4.H1814 11247796

[B43] Rodriguez-Cuenca S. Pellegrinelli V. Campbell M. Oresic M. Vidal-Puig A. (2017). Sphingolipids and glycerophospholipids-The “ying and yang” of lipotoxicity in metabolic diseases. Prog. Lipid Res. 66, 14–29. 10.1016/j.plipres.2017.01.002 28104532

[B44] Saddic L. A. Sigurdsson M. I. Chang T. W. Aranki S. F. Body S. C. Muehlschlegel J. D. (2017). The long noncoding RNA landscape of the ischemic human left ventricle. Circ. Cardiovasc. Genet. 10 (1), e001534. 10.1161/CIRCGENETICS.116.001534 28115490 PMC5302288

[B45] Sasset L. Zhang Y. Dunn T. M. Di Lorenzo A. (2016). Sphingolipid de novo biosynthesis: a rheostat of cardiovascular homeostasis. Trends Endocrinol. Metab. 27, 807–819. 10.1016/j.tem.2016.07.005 27562337 PMC5075255

[B46] Schiano C. Costa V. Aprile M. Donatelli F. Ciccodicola A. Napoli C. (2017). Heart failure: pilot transcriptomic analysis of cardiac tissue by RNA-sequencing. Cardiol. J. 24, 539–553. 10.5603/CJ.a2017.0052 28497843

[B47] Theilmeier G. Schmidt C. Herrmann J. Keul P. Chun J. Levkau B. (2006). High-density lipoproteins and their constituent, sphingosine-1-phosphate, directly protect the heart against ischemia/reperfusion injury *in vivo via the* S1P3 lysophospholipid receptor. Circulation. 114, 1403–1409. 10.1161/CIRCULATIONAHA.105.607135 16982942

[B48] Tragante V. Barnes M. R. Ganesh S. K. Munroe P. B. Keating B. J. (2014). Gene-centric meta-analysis in 87,736 individuals of European ancestry identifies multiple blood-pressure-related loci. Am. J. Hum. Genet. 94, 349–360. 10.1016/j.ajhg.2013.12.016 24560520 PMC3951943

[B49] Vausort M. Wagner D. R. Devaux Y. (2014). Long noncoding RNAs in patients with acute myocardial infarction. Circ. Res. 115, 668–677. 10.1161/CIRCRESAHA.115.303836 25035150

[B50] Vredevoe D. L. Moser D. K. Gan X. H. Bonavida B. (1995). Natural killer cell anergy to cytokine stimulants in a subgroup of patients with heart failure: relationship to norepinephrine. Neuroimmunomodulation. 2, 16–24. 10.1159/000096830 7614255

[B51] Wang Y. Jing W. Ma W. Liang C. Chai H. Tu J. (2018). Down-regulation of long non-coding RNA GAS5-AS1 and its prognostic and diagnostic significance in hepatocellular carcinoma. Cancer Biomark 22, 227–236. 10.3233/CBM-170781 29660898 PMC13078432

[B52] Wang Z. Zhang X. J. Ji Y. X. Zhang P. Chen I. Xiao X. (2016). The long noncoding RNA Chaer defines an epigenetic checkpoint in cardiac hypertrophy. Nat. Med. 22, 1131–1139. 10.1038/nm.4179 27618650 PMC5053883

[B53] Westman P. C. Lipinski M. J. Luger D. Waksman R. Bonow R. O. Wu E. (2016). Inflammation as a driver of adverse left ventricular remodeling after acute myocardial infarction. J. Am. Coll. Cardiol. 6, 2050–2060. 10.1016/j.jacc.2016.01.073 27126533

[B54] Yang K. C. Yamada K. A. Patel A. Y. George I. Mann D. L. Nerbonne J. M. (2014). Deep RNA sequencing reveals dynamic regulation of myocardial noncoding RNAs in failing human heart and remodeling with mechanical circulatory support. Circulation. 129, 1009–1021. 10.1161/CIRCULATIONAHA.113.003863 24429688 PMC3967509

[B55] Zhang X. Nie X. Yuan S. Hou H. Wang D. W. Chen C. (2019). Circulating long non-coding RNA ENST00000507296 is a prognostic indicator in patients with dilated cardiomyopathy. Mol. Ther. Nucleic Acids 16, 82–90. 10.1016/j.omtn.2019.02.004 30852379 PMC6409414

[B56] Zhou L. Huang H. McElfresh T. A. Prosdocimo D. A. Stanley W. C. (2008). Impact of anaerobic glycolysis and oxidative substrate selection on contractile function and mechanical efficiency during moderate severity ischemia. Am. J. Physiol. Heart Circ. Physiol. 95, 939–945. 10.1152/ajpheart.00561.2008 PMC2544504 18660443

