# Supplementary material for: Distinct Circulating Expression Profiles of Long Noncoding RNAs in Heart Failure Patients With Ischemic and Nonischemic Dilated Cardiomyopathy
Source: Front Genet. 2019 Nov 12;10:1116. doi: 10.3389/fgene.2019.01116 (PMC6861296; doi:10.3389/fgene.2019.01116)
Supplement: Supplementary file 1 [file DataSheet_1.docx]

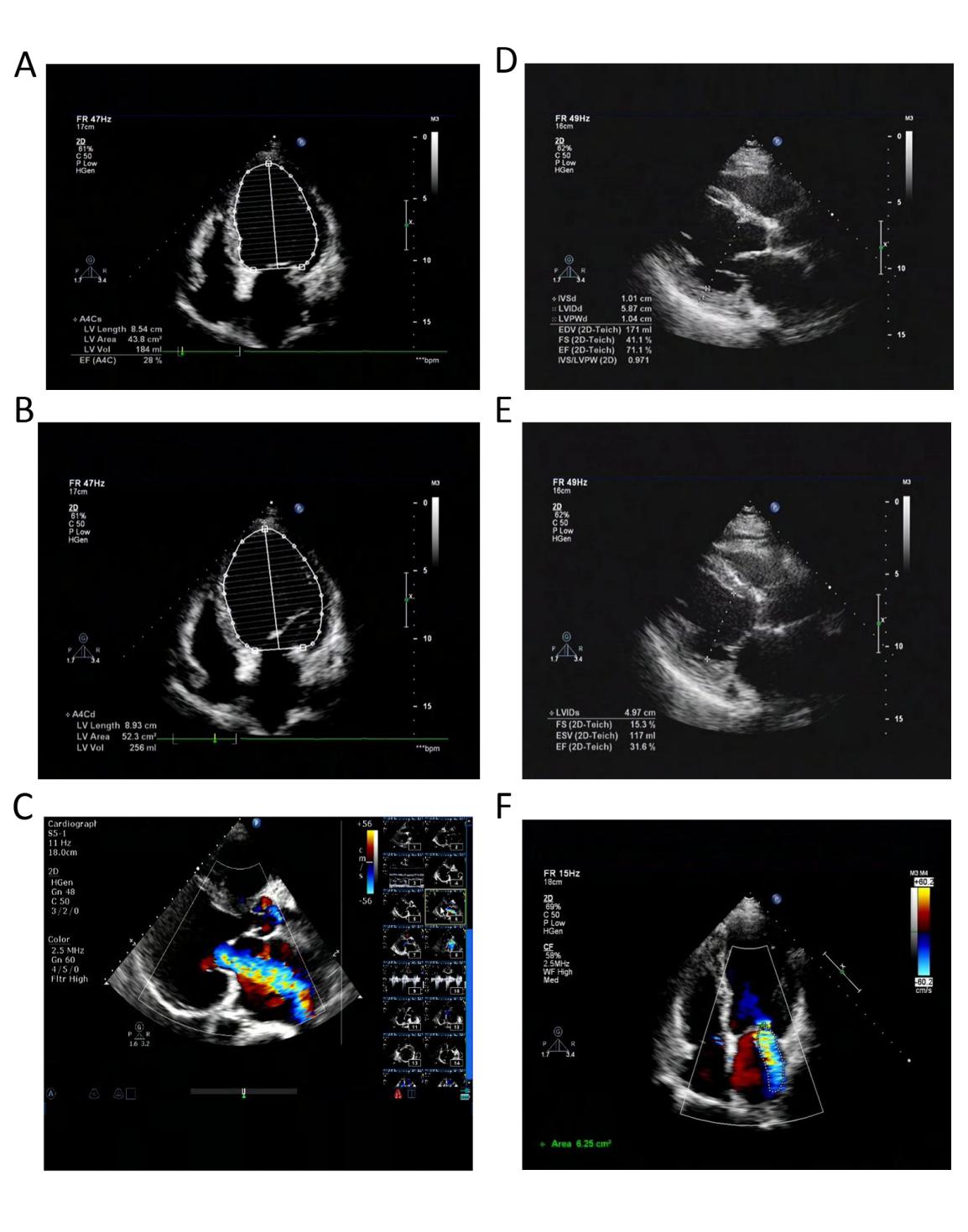


**Figure S1. Representative echocardiograms of DCM and ICM Patients.**

The typical echocardiograms of dilated cardiomyopathy (Fig S1A, S1B). In patients with dilated cardiomyopathy, left ventricular enlargement often leads to indirect enlargement of mitral annulus, resulting in mitral insufficiency (Fig S1C). The typical image of ischemic cardiomyopathy (FigS1D, S1E). Mitral regurgitation caused by papillary muscle dysfunction was also common in patients with ischemic cardiomyopathy (Fig S1F).

| **Sample ID** | **OD260/280 Ratio** | **OD260/230 Ratio** | **Conc. (ng/μl)** | **Volume (μl)** | **Quantity (ng)** | **QC result**  **Pass or Fail** |
| --- | --- | --- | --- | --- | --- | --- |
| DCM-1 | 1.74 | 1.22 | 214.89 | 15 | 3223.35 | Pass |
| DCM-2 | 1.68 | 1.14 | 190.67 | 15 | 2860.05 | Pass |
| DCM-3 | 1.44 | 0.75 | 203.97 | 15 | 3059.55 | Pass |
| DCM-4 | 1.68 | 1.46 | 167.10 | 15 | 2506.50 | Pass |
| DCM-5 | 1.68 | 0.98 | 206.62 | 15 | 3099.30 | Pass |
| DCM-6 | 1.74 | 2.02 | 105.83 | 15 | 1587.45 | Pass |
| DCM-7 | 1.72 | 1.26 | 136.60 | 15 | 2049.00 | Pass |
| DCM-8 | 1.72 | 1.56 | 128.99 | 15 | 1934.85 | Pass |
| DCM-9 | 1.43 | 0.81 | 78.38 | 15 | 1175.70 | Pass |
| DCM-10 | 1.68 | 1.52 | 132.31 | 15 | 1984.65 | Pass |
| DCM-11 | 1.68 | 1.39 | 157.80 | 15 | 2367.00 | Pass |
| ICM-1 | 1.72 | 1.55 | 144.38 | 15 | 2165.70 | Pass |
| ICM-2 | 1.55 | 0.90 | 217.44 | 15 | 3261.60 | Pass |
| ICM-3 | 1.65 | 1.26 | 127.44 | 15 | 1911.60 | Pass |
| ICM-4 | 1.59 | 1.42 | 117.41 | 15 | 1761.15 | Pass |
| ICM-5 | 1.73 | 1.43 | 206.30 | 15 | 3094.50 | Pass |
| ICM-6 | 1.79 | 1.62 | 209.11 | 15 | 3136.65 | Pass |
| ICM-7 | 1.68 | 1.69 | 110.74 | 15 | 1661.10 | Pass |
| ICM-8 | 1.64 | 1.71 | 102.45 | 15 | 1536.75 | Pass |
| ICM-9 | 1.51 | 0.84 | 206.70 | 15 | 3100.50 | Pass |

**Table S1. The quality and quantity of extracted RNA**

**Table S2. Top 20 aberrantly up-regulated lncRNAs in microarray analysis.**

| Seq  ID | *P* value | Fold  Change | Regulation | Chr | Strand | Relationship | DCM  -1 | DCM  -2 | DCM  -3 | DCM  -4 | DCM  -5 | DCM  -6 | DCM  -7 | DCM  -8 | DCM  -9 | DCM  -10 | DCM  -11 | ICM  -1 | ICM  -2 | ICM  -3 | ICM  -4 | ICM  -5 | ICM  -6 | ICM  -7 | ICM-8 | ICM-9 |
| --- | --- | --- | --- | --- | --- | --- | --- | --- | --- | --- | --- | --- | --- | --- | --- | --- | --- | --- | --- | --- | --- | --- | --- | --- | --- | --- |
| T342171 | 1.34894E-08 | 231.48612 | Up | Chr 8 | - | intergenic | 13.50589 | 12.95858 | 13.75998 | 13.72479 | 12.87608 | 12.12510 | 12.37757 | 11.29148 | 13.09163 | 12.84360 | 10.72855 | 3.08762 | 2.33440 | 7.46859 | 2.43515 | 8.40372 | 4.82937 | 2.33270 | 4.77686 | 7.59757 |
| T251871 | 1.19729E-08 | 150.31172 | up | Chr 3 | - | intergenic | 10.19270 | 12.16515 | 12.04906 | 12.62987 | 11.01198 | 9.27480 | 9.68854 | 7.64822 | 11.09315 | 10.14975 | 6.84822 | 2.34242 | 2.33440 | 2.34294 | 5.15444 | 2.32668 | 2.32395 | 5.67940 | 2.33583 | 2.32478 |
| T207073 | 6.05435E-06 | 97.37735 | up | Chr 2 | - | intergenic | 8.07620 | 8.52674 | 11.63165 | 12.08038 | 10.99708 | 10.50562 | 10.90271 | 3.85113 | 11.00863 | 8.27650 | 2.73190 | 2.34242 | 2.33440 | 2.34294 | 2.32296 | 2.32668 | 2.32395 | 2.33270 | 2.33583 | 2.55184 |
| T167195 | 3.18310E-06 | 86.83382 | up | Chr 19 | + | intergenic | 10.64852 | 11.69754 | 12.15484 | 12.67386 | 11.12974 | 8.41655 | 8.69826 | 8.48161 | 11.36245 | 11.02116 | 8.08168 | 2.34242 | 2.94317 | 3.59261 | 8.20257 | 2.61046 | 2.32395 | 8.93489 | 2.33583 | 2.32479 |
| T095115 | 1.81297E-06 | 76.63745 | up | Chr 13 | + | intergenic | 8.61644 | 10.39452 | 12.76397 | 12.71769 | 11.01382 | 9.88290 | 9.98616 | 6.45297 | 10.65500 | 7.80965 | 7.86149 | 2.34242 | 2.33440 | 2.34294 | 3.48279 | 7.14804 | 2.32395 | 2.84094 | 2.33583 | 6.99902 |
| T260785 | 1.23707E-05 | 74.83282 | up | Chr 4 | - | intergenic | 5.34086 | 5.35647 | 6.08168 | 6.28848 | 12.68448 | 11.76771 | 11.90187 | 9.90573 | 13.08375 | 8.92077 | 10.35063 | 4.71936 | 2.33440 | 2.34294 | 3.26873 | 2.55977 | 2.32395 | 2.33270 | 4.23847 | 3.04399 |
| T365797 | 4.55300E-10 | 73.27723 | up | Chr 9 | - | intergenic | 12.29403 | 9.25911 | 9.67819 | 8.90488 | 9.24997 | 11.50204 | 11.43879 | 11.52900 | 9.00737 | 12.22474 | 11.77668 | 4.97582 | 3.16688 | 3.55849 | 4.62087 | 4.60531 | 5.48658 | 4.22197 | 5.03193 | 4.19116 |
| T133054 | 6.07700E-06 | 66.11780 | up | Chr 16 | + | intergenic | 7.53074 | 12.02933 | 12.51385 | 12.50786 | 11.58944 | 11.24852 | 10.80770 | 11.47468 | 11.41964 | 9.12094 | 12.18958 | 3.72495 | 2.33440 | 2.34294 | 7.87489 | 9.41585 | 2.97459 | 6.41676 | 3.22564 | 7.43915 |
| T281902 | 4.06290E-09 | 65.55324 | up | Chr 5 | - | intergenic | 8.40126 | 7.19085 | 7.46680 | 7.81703 | 11.34211 | 10.10109 | 10.23911 | 8.79376 | 11.68905 | 10.35063 | 9.47005 | 3.08305 | 2.33440 | 3.32442 | 4.20494 | 2.32668 | 4.71976 | 2.90479 | 4.09006 | 2.86015 |
| T360865 | 6.46965E-05 | 64.41642 | up | Chr 9 | - | intergenic | 10.09597 | 11.76648 | 12.04525 | 12.48857 | 10.88299 | 9.63541 | 9.78558 | 3.95957 | 11.03884 | 10.36275 | 3.33294 | 3.00550 | 2.33440 | 3.48966 | 6.73757 | 2.32668 | 3.02210 | 6.57100 | 2.33583 | 2.32479 |
| TCONS_00021582 | 1.37227E-04 | 62.74320 | up | Chr 13 | + | intergenic | 6.80265 | 11.40489 | 12.69201 | 12.70427 | 11.25048 | 10.99708 | 10.99153 | 3.76800 | 11.19437 | 7.40242 | 4.15332 | 2.34242 | 2.33440 | 2.34294 | 7.17090 | 2.32668 | 2.57969 | 6.32987 | 3.07393 | 2.32479 |
| T344979 | 1.16162E-04 | 62.54691 | up | Chr 8 | + | intergenic | 3.90433 | 12.26346 | 12.79993 | 13.13786 | 11.61846 | 9.46923 | 9.70222 | 7.78571 | 11.29346 | 7.08203 | 7.86902 | 2.34242 | 2.33440 | 2.34294 | 3.53672 | 8.55254 | 2.32395 | 2.33270 | 2.33583 | 7.68137 |
| NR_003569 | 1.75236E-07 | 60.61788 | up | Chr 16 | + | intergenic | 10.23378 | 6.90451 | 10.28006 | 10.04026 | 9.84991 | 10.40409 | 9.84430 | 6.00858 | 9.25839 | 9.99549 | 6.70720 | 2.34242 | 2.33440 | 6.91121 | 2.32296 | 4.21342 | 2.32395 | 2.33270 | 2.33583 | 3.01889 |
| T191270 | 3.65876E-07 | 60.20377 | up | Chr 2 | + | intergenic | 9.68216 | 8.02589 | 10.75599 | 10.88093 | 9.18672 | 6.89869 | 7.04055 | 4.37276 | 9.23253 | 9.96871 | 4.63502 | 2.34242 | 2.33440 | 2.34294 | 2.32296 | 2.32668 | 2.32395 | 2.33270 | 2.33583 | 2.32479 |
| T210074 | 5.48178E-05 | 53.39808 | up | Chr 2 | + | intergenic | 2.32623 | 4.23907 | 11.18467 | 11.27887 | 9.30401 | 9.66057 | 10.08581 | 10.05598 | 9.21521 | 3.56287 | 10.18613 | 2.34242 | 2.33440 | 2.34294 | 2.38282 | 2.32668 | 3.09990 | 2.33270 | 3.40079 | 2.32479 |
| T226011 | 1.15775E-08 | 52.26800 | up | Chr 21 | - | intergenic | 8.59452 | 6.33444 | 7.77341 | 7.47656 | 10.30228 | 9.58026 | 8.72938 | 4.95674 | 10.00930 | 8.77014 | 5.90974 | 2.34242 | 2.33440 | 2.34294 | 2.32296 | 2.32668 | 2.32395 | 2.33270 | 2.33583 | 2.32479 |
| T102207 | 4.70837E-06 | 52.22485 | up | Chr 14 | + | intergenic | 8.34417 | 10.37997 | 8.88116 | 9.72882 | 9.38850 | 10.04860 | 10.08176 | 2.99502 | 9.59115 | 9.73353 | 3.99500 | 2.34242 | 2.33440 | 2.34294 | 4.90034 | 2.32668 | 2.69373 | 3.26698 | 2.33583 | 2.32479 |
| TCONS_00005118 | 3.54561E-05 | 48.73861 | up | Chr 2 | - | intergenic | 11.11700 | 4.33739 | 11.90678 | 12.16946 | 10.10691 | 6.31273 | 6.01138 | 5.37636 | 10.09062 | 10.63551 | 5.00958 | 2.34242 | 3.58733 | 2.34294 | 2.53348 | 2.65448 | 3.22636 | 2.33270 | 3.79453 | 2.87405 |
| uc.417- | 6.21178E-08 | 48.61328 | up | Chr 17 | - | exon sense-overlapping | 10.61752 | 11.78189 | 11.92571 | 12.33331 | 11.58502 | 10.13845 | 10.65196 | 9.73780 | 11.58146 | 11.02632 | 9.39734 | 4.90246 | 2.33440 | 6.65022 | 4.15181 | 7.40439 | 7.45508 | 4.00547 | 4.34539 | 7.13861 |
| ENST00000521696 | 1.67434E-06 | 48.51561 | up | Chr 8 | - | bidirectional | 8.58862 | 13.42845 | 11.29733 | 11.23968 | 12.03980 | 11.44763 | 11.62781 | 10.62848 | 11.78808 | 8.99474 | 10.24115 | 5.35056 | 4.35408 | 11.08367 | 4.74269 | 3.99268 | 5.65947 | 4.60033 | 5.16892 | 3.90748 |

**Table S2. Top 20 aberrantly up-regulated lncRNAs in microarray analysis.** SeqID: lncRNA name. P value: P value calculated from unpaired t-test. Fold Change: the absolute ratio (no log scale) of normalized intensities between two groups(DCM vs ICM). Chr: chromosome number from which lncRNA is transcribed. Strand: the strand of chromosome from which the lncRNA is transcribed; “+” is the sense strand of the chromosome, “−” is the antisense strand of the chromosome. Relationship: intergenic, there are no coding transcripts within 30 kb of the lncRNA; bidirectional, RNA molecules that are oriented head to head to a coding transcript within 1000 bp; exon sense-overlapping: RNA molecules transcribed from the antisense strand and overlapping with a coding transcript. ICM 1-9 and DCM1-11: normalized intensity of each sample (log2 transformed)

**Table S3. Top 20 aberrantly down-regulated lncRNAs in microarray analysis.**

| Seq  ID | *P* value | Fold  Change | Regulation | Chr | Strand | Relationship | DCM  -1 | DCM  -2 | DCM  -3 | DCM  -4 | DCM  -5 | DCM  -6 | DCM  -7 | DCM  -8 | DCM  -9 | DCM  -10 | DCM  -11 | ICM  -1 | ICM  -2 | ICM  -3 | ICM  -4 | ICM  -5 | ICM  -6 | ICM  -7 | ICM  -8 | ICM  -9 |
| --- | --- | --- | --- | --- | --- | --- | --- | --- | --- | --- | --- | --- | --- | --- | --- | --- | --- | --- | --- | --- | --- | --- | --- | --- | --- | --- |
| ENST00000445280 | 4.25714E-06 | 66.66290 | down | chr9 | - | intergenic | 2.32623 | 9.62829 | 2.33888 | 2.33029 | 4.57964 | 5.47651 | 6.18342 | 4.14794 | 4.48586 | 2.73381 | 3.95341 | 11.93594 | 6.11659 | 9.15196 | 10.83104 | 9.38850 | 12.19064 | 11.32572 | 11.87633 | 11.13609 |
| T181556 | 2.21981E-05 | 59.60329 | down | chr19 | - | intergenic | 2.62500 | 10.30555 | 2.33888 | 2.33029 | 6.32465 | 5.58816 | 6.12877 | 4.78440 | 6.02450 | 2.39850 | 4.81096 | 11.90795 | 5.59292 | 9.33674 | 12.02810 | 10.60506 | 11.89187 | 12.25144 | 12.08147 | 11.28370 |
| ENST00000472513 | 4.62918E-03 | 58.19568 | down | chr3 | + | intronic antisense | 2.32623 | 2.33005 | 2.33888 | 2.33029 | 2.32162 | 2.35995 | 2.79561 | 2.32649 | 2.32106 | 2.37194 | 2.32451 | 14.63637 | 2.33440 | 2.34294 | 3.70822 | 3.10350 | 14.98579 | 4.33789 | 14.54624 | 14.16290 |
| T380669 | 6.35649E-05 | 57.03243 | down | chrX | - | intergenic | 2.52117 | 10.61862 | 2.33888 | 2.58890 | 3.28717 | 5.49385 | 6.22400 | 4.59025 | 3.20584 | 2.38117 | 4.29297 | 11.81555 | 4.79619 | 6.79725 | 11.02515 | 10.63164 | 11.58872 | 11.45283 | 12.02933 | 11.26539 |
| ENST00000597337 | 1.63861E-05 | 56.68012 | down | chr19 | - | natural antisense | 6.46869 | 12.64329 | 2.33888 | 2.33029 | 6.68368 | 7.98797 | 8.50478 | 6.48164 | 6.46948 | 5.78974 | 5.96215 | 13.38849 | 10.08075 | 11.21334 | 12.51091 | 11.81052 | 13.13488 | 12.65273 | 13.25853 | 13.00417 |
| T379563 | 2.77328E-05 | 48.28177 | down | chrX | - | intergenic | 2.52768 | 9.68018 | 2.33888 | 2.33029 | 4.39521 | 4.86618 | 5.11201 | 4.21504 | 3.99832 | 2.43167 | 3.43878 | 10.92042 | 3.85663 | 8.14003 | 10.77650 | 10.33601 | 10.68945 | 11.15641 | 11.04143 | 10.51543 |
| T256902 | 3.48615E-05 | 46.91170 | down | chr3 | - | intergenic | 2.49262 | 9.81225 | 2.33888 | 2.33029 | 4.38705 | 4.16877 | 4.58963 | 3.85430 | 4.38641 | 3.58269 | 2.92609 | 10.89737 | 3.86736 | 7.47878 | 11.13890 | 9.75876 | 10.93857 | 11.29733 | 11.06044 | 10.24036 |
| T198560 | 7.64961E-06 | 44.29688 | down | chr2 | + | bidirectional | 2.32623 | 8.63815 | 2.33888 | 2.33029 | 4.07308 | 4.43102 | 5.12074 | 3.04806 | 3.74175 | 3.62117 | 2.32451 | 10.85975 | 5.07724 | 7.64896 | 9.89867 | 7.51332 | 11.20777 | 10.54547 | 10.89737 | 9.93227 |
| ENST00000428188 | 8.23775E-05 | 43.37516 | down | chr2 | - | intronic antisense | 2.32623 | 9.45763 | 2.33888 | 2.33029 | 2.81593 | 4.16312 | 4.41191 | 3.77800 | 2.69931 | 2.32208 | 3.76242 | 10.85410 | 3.94483 | 4.80354 | 10.53822 | 9.54064 | 10.33429 | 10.98931 | 11.03208 | 9.97146 |
| T042208 | 6.12542E-05 | 38.05197 | down | chr10 | + | intergenic | 2.32623 | 9.39230 | 2.33888 | 2.33029 | 4.01571 | 4.67967 | 5.47377 | 3.69832 | 3.73409 | 2.32208 | 3.40324 | 10.42452 | 3.44154 | 7.19017 | 10.38082 | 9.55197 | 10.48830 | 10.98126 | 10.66979 | 9.88718 |
| T280062 | 6.32169E-05 | 35.23176 | down | chr5 | - | intergenic | 2.32623 | 9.27698 | 2.33888 | 2.33029 | 4.26334 | 4.84097 | 5.61819 | 4.56018 | 4.30871 | 2.42110 | 3.82435 | 10.94045 | 3.69383 | 8.27394 | 10.12147 | 8.22594 | 11.32300 | 10.67693 | 10.80653 | 9.91286 |
| ENST00000527239 | 2.82349E-04 | 24.87700 | down | chr11 | - | intergenic | 2.32623 | 8.84450 | 2.33888 | 2.57558 | 2.40856 | 3.70765 | 4.43987 | 2.62617 | 2.67305 | 2.32208 | 3.03786 | 9.26196 | 2.33440 | 4.86021 | 9.89664 | 8.05129 | 9.51033 | 10.45594 | 9.47005 | 8.40837 |
| NR_026677 | 2.46960E-04 | 24.54583 | down | chr9 | - | intergenic | 2.32623 | 3.15045 | 2.33888 | 3.74868 | 5.06222 | 2.38269 | 3.03177 | 4.68390 | 4.93899 | 2.38785 | 4.40542 | 10.28104 | 3.91236 | 2.34294 | 10.09532 | 6.17855 | 10.42182 | 10.32696 | 10.25449 | 9.20806 |
| TCONS_00019584 | 1.57830E-05 | 23.41880 | down | chr11 | - | intergenic | 5.33714 | 9.16602 | 3.30417 | 3.54449 | 5.05254 | 5.85524 | 6.26930 | 5.80264 | 5.15354 | 5.45354 | 6.15469 | 11.41744 | 5.83001 | 8.49370 | 11.04204 | 9.08487 | 11.68307 | 11.32459 | 11.41964 | 10.63645 |
| T252655 | 4.03326E-04 | 20.99758 | down | chr3 | + | intergenic | 7.18066 | 2.33005 | 2.33888 | 2.33029 | 2.32162 | 4.28259 | 4.51099 | 7.12059 | 2.86104 | 8.07012 | 7.21599 | 10.82876 | 7.92427 | 6.49139 | 6.55627 | 9.74858 | 11.56811 | 6.49461 | 11.11013 | 10.17684 |
| GSE61474_TCONS_00130329 | 4.51441E-03 | 18.52061 | down | chr16 | - | intergenic | 4.47844 | 5.63463 | 2.33888 | 3.19043 | 7.82770 | 4.64095 | 4.19184 | 3.20958 | 7.75110 | 4.25194 | 3.63395 | 11.46848 | 12.26492 | 2.34294 | 9.03462 | 2.32668 | 11.30556 | 9.22936 | 11.45509 | 10.32143 |
| T132397 | 4.42397E-03 | 18.28868 | down | chr16 | + | intergenic | 11.21430 | 4.09994 | 3.27172 | 2.33029 | 2.56127 | 8.40681 | 8.58561 | 9.31574 | 2.57655 | 11.90678 | 9.36376 | 10.90070 | 10.29360 | 8.93982 | 10.30161 | 12.49356 | 11.73099 | 10.57431 | 11.13609 | 11.61021 |
| ENST00000464125 | 5.34950E-05 | 18.24752 | down | chr3 | + | intronic antisense | 3.63713 | 9.02148 | 2.33888 | 2.33029 | 2.53732 | 4.55319 | 5.02927 | 3.88015 | 2.65107 | 3.71441 | 3.19213 | 8.16045 | 4.99461 | 6.20264 | 9.51106 | 9.07755 | 8.50800 | 9.93423 | 8.13792 | 8.26819 |
| uc001oou.3 | 8.39120E-05 | 15.60205 | down | chr11 | - | natural antisense | 2.32623 | 8.26965 | 2.33888 | 2.33029 | 2.45928 | 3.86736 | 4.44571 | 3.09818 | 2.32106 | 2.32208 | 2.57076 | 7.59263 | 4.20836 | 4.82677 | 8.50862 | 7.97439 | 8.29590 | 9.28747 | 7.30786 | 7.41146 |
| TCONS_00006918 | 2.01136E-02 | 14.50626 | down | chr3 | + | natural antisense | 12.21191 | 4.19154 | 2.88907 | 3.29629 | 8.81512 | 13.68714 | 13.93715 | 13.72917 | 8.73566 | 12.54413 | 13.33706 | 12.87608 | 13.75256 | 12.23342 | 14.03936 | 14.97432 | 13.04796 | 14.32974 | 12.76713 | 14.55853 |

**Table S3. Top 20 aberrantly down-regulated lncRNAs in microarray analysis.** SeqID: lncRNA name. P value: P value calculated from unpaired t-test. Fold Change: the absolute ratio (no log scale) of normalized intensities between two groups(DCM vs ICM). Chr: chromosome number from which lncRNA is transcribed. Strand: the strand of chromosome from which the lncRNA is transcribed; “+” is the sense strand of the chromosome, “−” is the antisense strand of the chromosome. Intergenic, there are no coding transcripts within 30 kb of the lncRNA; Intronic antisense, RNA molecules that are transcribed from the antisense strand without sharing overlapping exons; Natural antisense, RNA molecules transcribed from the antisense strand and overlapping in part with well-defined spliced sense or intronless sense RNAs; Bidirectional, RNA molecules that are oriented head to head to a coding transcript within 1000 bp. DCM 1-11 and ICM1-9: normalized intensity of each sample (log2 transformed)

**Table S4. Top 20 aberrantly up-regulated mRNAs in microarray analysis.**

| Seqname | Gene  Symblo | *P* value | Fold  Change | Regulation | Chr | Strand | DCM  -1 | DCM  -2 | DCM  -3 | DCM  -4 | DCM  -5 | DCM  -6 | DCM  -7 | DCM  -8 | DCM  -9 | DCM  -10 | DCM  -11 | ICM  -1 | ICM  -2 | ICM  -3 | ICM  -4 | ICM  -5 | ICM  -6 | ICM  -7 | ICM  -8 | ICM  -9 |
| --- | --- | --- | --- | --- | --- | --- | --- | --- | --- | --- | --- | --- | --- | --- | --- | --- | --- | --- | --- | --- | --- | --- | --- | --- | --- | --- |
| NM_145059 | FUK | 4.10000E-12 | 216.16425 | Up | chr16 | + | 11.69317 | 10.21248 | 11.70834 | 11.73434 | 11.10570 | 10.20916 | 10.25556 | 9.58105 | 10.76853 | 11.33184 | 9.64607 | 2.34242 | 2.33440 | 2.34294 | 2.32296 | 5.74485 | 2.32395 | 2.33270 | 2.33583 | 4.86304 |
| NM_006136 | CAPZA2 | 3.88116E-05 | 126.73961 | up | chr7 | + | 11.62089 | 13.05256 | 13.43519 | 13.89481 | 12.58815 | 10.60408 | 11.33944 | 4.47805 | 12.62526 | 11.38340 | 4.07710 | 2.34242 | 2.33440 | 5.29689 | 6.81499 | 2.32668 | 3.53921 | 7.25784 | 2.33583 | 2.32479 |
| NM_014569 | ZKSCAN5 | 1.00000E-12 | 103.19218 | up | chr7 | + | 9.88718 | 8.94301 | 10.73324 | 10.82540 | 8.90581 | 8.65858 | 7.70296 | 9.65273 | 8.73258 | 9.65960 | 9.83553 | 2.34242 | 2.33440 | 4.55856 | 3.13618 | 2.82024 | 2.32395 | 2.33270 | 2.33583 | 2.32479 |
| NM_006110 | CD2BP2 | 9.20155E-07 | 98.33423 | up | chr16 | - | 9.43372 | 12.40669 | 6.13096 | 6.24069 | 11.10409 | 10.48033 | 10.60056 | 10.01481 | 10.78995 | 8.96209 | 10.09532 | 2.34242 | 2.33440 | 8.71903 | 2.32296 | 2.32668 | 2.32395 | 2.33270 | 2.33583 | 2.32479 |
| NM_001159279 | ZNF716 | 3.77300E-08 | 77.36574 | up | chr7 | + | 6.99959 | 11.97834 | 6.71402 | 6.01138 | 10.70910 | 9.65776 | 9.76890 | 9.53895 | 10.22963 | 6.68643 | 9.56131 | 2.34242 | 2.33440 | 4.50256 | 2.67489 | 2.32668 | 2.32395 | 2.33270 | 2.33583 | 2.42748 |
| NM_002172 | IFNA14 | 5.75589E-06 | 70.37232 | up | chr9 | - | 2.72029 | 8.62224 | 11.43403 | 11.84947 | 10.22237 | 9.85961 | 9.92186 | 7.70066 | 10.21675 | 4.14629 | 7.79435 | 3.19900 | 2.33440 | 2.34294 | 2.32296 | 2.32668 | 2.32395 | 2.33270 | 2.33583 | 2.55742 |
| NM_001009992 | ZNF648 | 2.74065E-06 | 65.25381 | up | chr1 | - | 4.72189 | 12.20189 | 6.91246 | 6.65477 | 11.18777 | 10.05080 | 10.12065 | 9.44529 | 10.93332 | 5.07549 | 9.44158 | 2.34242 | 2.33440 | 5.25676 | 2.41885 | 2.32668 | 2.57421 | 2.33270 | 2.99302 | 2.32479 |
| NM_014600 | EHD3 | 6.10184E-07 | 58.88600 | up | chr2 | + | 11.07231 | 2.72840 | 9.13289 | 9.34141 | 7.97311 | 9.74417 | 9.95268 | 9.12954 | 7.99305 | 10.73974 | 9.15298 | 2.34242 | 2.33440 | 2.91189 | 4.27382 | 3.50630 | 2.32395 | 3.94414 | 2.33583 | 2.43972 |
| NM_013400 | REPIN1 | 8.99980E-09 | 53.44164 | up | chr7 | + | 10.96677 | 10.64972 | 11.62089 | 12.02810 | 11.14619 | 9.67651 | 9.29594 | 9.56803 | 11.35880 | 10.92604 | 9.24357 | 2.34242 | 3.98811 | 7.09904 | 6.26466 | 5.97215 | 4.06762 | 5.58726 | 3.09472 | 5.22724 |
| NM_007215 | POLG2 | 1.37220E-04 | 51.40489 | up | chr17 | - | 6.85693 | 8.43183 | 12.91086 | 12.77129 | 10.38338 | 8.15500 | 6.96365 | 2.32649 | 10.04328 | 7.72879 | 2.32451 | 2.34242 | 2.33440 | 2.34294 | 2.49002 | 2.32668 | 2.74880 | 2.33270 | 2.33583 | 2.32479 |
| NM_001159524 | ZNF735 | 2.51532E-06 | 50.64879 | up | chr7 | + | 5.24085 | 11.78901 | 5.77197 | 4.74632 | 10.18913 | 9.32615 | 9.42810 | 9.01474 | 9.85088 | 4.87393 | 9.13373 | 2.34242 | 2.33440 | 3.51085 | 2.32296 | 2.32668 | 2.32395 | 2.33270 | 2.33583 | 2.32479 |
| NM_016458 | HGH1 | 8.28897E-04 | 50.13938 | up | chr8 | + | 12.49225 | 4.22346 | 11.19347 | 11.45779 | 12.24495 | 6.24774 | 6.25293 | 2.79907 | 12.72253 | 11.79848 | 2.48649 | 2.34242 | 2.33440 | 2.34294 | 2.32296 | 4.68669 | 2.32395 | 2.79945 | 3.50443 | 3.35488 |
| NM_018289 | VPS53 | 4.31000E-11 | 49.10904 | up | chr17 | - | 9.23017 | 9.33437 | 8.51992 | 8.46805 | 9.15913 | 8.62025 | 8.69142 | 6.22725 | 8.81512 | 8.43819 | 6.12832 | 2.34242 | 3.86762 | 2.95883 | 2.39738 | 2.32668 | 2.93074 | 2.33270 | 2.33583 | 2.91833 |
| NM_133466 | ZFP82 | 1.24126E-06 | 47.52475 | up | chr19 | - | 10.76970 | 10.83668 | 11.32193 | 11.33184 | 9.63785 | 9.44981 | 8.83481 | 5.67349 | 9.50422 | 10.62128 | 6.53721 | 4.68988 | 2.33440 | 2.34294 | 2.59175 | 6.44429 | 4.85577 | 2.33270 | 4.48285 | 5.30536 |
| NM_001714 | BICD1 | 1.43571E-04 | 46.35783 | up | chr12 | + | 2.32623 | 5.07166 | 8.94301 | 8.28744 | 10.64729 | 12.02560 | 12.12510 | 7.72736 | 10.51716 | 2.74455 | 9.13577 | 2.34242 | 2.33440 | 3.03092 | 2.39478 | 3.45720 | 2.32395 | 2.33270 | 2.91531 | 2.32479 |
| NM_030915 | LBH | 9.33590E-06 | 45.67550 | up | chr2 | + | 9.24912 | 12.48077 | 10.44668 | 10.73324 | 11.32193 | 8.65020 | 9.69724 | 9.25741 | 11.21879 | 8.83818 | 7.92625 | 2.85614 | 3.06538 | 10.98679 | 4.83802 | 4.21004 | 2.42100 | 4.98338 | 3.03451 | 3.83718 |
| NM_032421 | CLIP2 | 1.83205E-04 | 42.07722 | up | chr7 | + | 10.24720 | 11.17947 | 9.59862 | 9.14335 | 11.91488 | 10.72635 | 10.98508 | 11.51701 | 11.67570 | 10.28006 | 11.26875 | 2.34242 | 2.33440 | 2.34294 | 7.77060 | 10.62848 | 2.32395 | 8.19870 | 2.33583 | 10.15236 |
| NM_004407 | DMP1 | 2.41934E-07 | 41.86692 | up | chr4 | + | 9.33346 | 12.09352 | 6.90270 | 6.55867 | 10.83440 | 10.56643 | 10.48224 | 9.89664 | 10.75921 | 8.71819 | 10.00107 | 2.34242 | 4.78357 | 6.15586 | 4.96377 | 4.98543 | 2.51120 | 4.65688 | 3.92680 | 4.03159 |
| NM_021057 | IFNA7 | 3.69190E-05 | 40.84990 | up | chr9 | - | 2.32623 | 7.94583 | 9.03909 | 9.01329 | 9.54797 | 9.46485 | 8.83166 | 10.06845 | 9.41493 | 2.36775 | 10.33061 | 2.34242 | 2.33440 | 2.34294 | 4.29335 | 2.32668 | 2.32395 | 3.19757 | 2.33583 | 2.61940 |
| NM_018234 | STEAP3 | 1.73182E-04 | 40.59602 | up | chr2 | + | 9.54733 | 12.98251 | 8.81448 | 9.05353 | 9.44782 | 4.68619 | 4.56329 | 2.55693 | 9.42223 | 10.32390 | 3.02810 | 2.34242 | 2.33440 | 2.34294 | 2.32296 | 2.32668 | 2.32395 | 2.33270 | 2.33583 | 2.32479 |

**Table S4. Top 20 aberrantly up-regulated mRNAs in microarray analysis.** Seqname: sequence name. Gene symbol: gene name. P value: P value calculated from unpaired t-test. Fold change: the absolute ratio (no log scale) of normalized intensities between two groups (DCM vs ICM). Chr: chromosome number from which the mRNA is transcribed. Strand: the strand of chromosome from which the mRNA is transcribed; “+” is the sense strand of the chromosome, “−” is the antisense strand of the chromosome .DCM 1-11 and ICM1-9: normalized intensity of each sample (log2 transformed)

**Table S5. Top 20 aberrantly down-regulated mRNAs in microarray analysis.**

| Seqname | Gene  Symblo | *P* value | Fold  Change | Regulation | Chr | Strand | DCM  -1 | DCM  -2 | DCM  -3 | DCM  -4 | DCM  -5 | DCM  -6 | DCM  -7 | DCM  -8 | DCM  -9 | DCM  -10 | DCM  -11 | ICM  -1 | ICM  -2 | ICM  -3 | ICM  -4 | ICM  -5 | ICM  -6 | ICM  -7 | ICM  -8 | ICM  -9 |
| --- | --- | --- | --- | --- | --- | --- | --- | --- | --- | --- | --- | --- | --- | --- | --- | --- | --- | --- | --- | --- | --- | --- | --- | --- | --- | --- |
| NM_003042 | SLC6A1 | 2.73769E-03 | 18.49104 | down | chr3 | + | 5.99576 | 3.92902 | 3.89728 | 3.88724 | 3.74140 | 3.54734 | 3.49366 | 10.59756 | 3.74140 | 5.54565 | 10.34040 | 11.80819 | 12.81485 | 7.26664 | 6.46506 | 6.39979 | 11.82317 | 6.73532 | 11.77892 | 10.82778 |
| NM_018348 | CMTR2 | 2.14849E-04 | 17.27547 | down | chr16 | - | 2.32623 | 7.48120 | 2.33888 | 2.33029 | 2.44398 | 3.09928 | 3.91705 | 2.32649 | 2.37431 | 2.32208 | 2.32451 | 8.82691 | 2.33440 | 4.57109 | 8.16467 | 5.90235 | 9.53383 | 8.51393 | 9.02633 | 7.35497 |
| NM_000145 | FSHR | 2.68578E-03 | 15.50589 | down | chr2 | - | 5.58295 | 5.69129 | 7.59684 | 7.21418 | 10.28468 | 9.98513 | 9.79252 | 2.32649 | 10.49454 | 5.02036 | 2.60204 | 12.60660 | 10.88299 | 6.70993 | 10.49454 | 10.44613 | 12.37154 | 10.49580 | 12.37424 | 11.87633 |
| NM_003053 | SLC18A1 | 3.19455E-03 | 15.45692 | down | chr8 | - | 3.01866 | 3.43735 | 3.82245 | 3.53727 | 8.79477 | 6.46696 | 6.45062 | 2.32649 | 8.97685 | 2.61617 | 2.32451 | 11.30240 | 9.66544 | 3.40820 | 6.88067 | 7.25526 | 11.13212 | 7.21418 | 11.23740 | 9.81494 |
| NM_145236 | B3GNT7 | 1.19524E-03 | 14.27451 | down | chr2 | + | 5.92264 | 6.47809 | 6.49589 | 6.76406 | 5.82223 | 10.43625 | 10.45367 | 6.74696 | 5.60430 | 5.33088 | 7.04556 | 13.07610 | 12.19064 | 6.33030 | 10.21248 | 6.85395 | 13.14290 | 10.18302 | 13.15171 | 12.45948 |
| NM_033081 | DIDO1 | 7.35732E-03 | 12.80703 | down | chr20 | - | 4.87263 | 2.98752 | 2.75200 | 2.33029 | 2.63109 | 2.61173 | 2.93199 | 2.88453 | 2.90981 | 5.42443 | 3.22245 | 11.22978 | 3.35908 | 3.16103 | 3.25411 | 5.27016 | 11.28736 | 3.27494 | 11.08468 | 10.28193 |
| NM_012231 | PRDM2 | 9.84650E-04 | 12.77599 | down | chr1 | + | 2.50774 | 2.33005 | 3.56906 | 2.33029 | 3.19730 | 2.56722 | 2.50076 | 7.13411 | 3.56349 | 2.32208 | 7.28612 | 9.31894 | 9.08487 | 8.93842 | 4.84320 | 3.05231 | 7.79668 | 5.18130 | 9.44713 | 7.57670 |
| NM_004583 | RAB5C | 1.72883E-02 | 12.55324 | down | chr17 | - | 8.86682 | 2.33005 | 2.33888 | 2.33029 | 9.21330 | 7.81829 | 7.72084 | 10.71398 | 9.52229 | 9.42223 | 10.65316 | 12.42968 | 12.40979 | 10.80023 | 11.16897 | 3.93623 | 12.69201 | 11.29640 | 12.43608 | 11.89605 |
| NM_001242901 | DPP9-AS1 | 1.79156E-05 | 12.44411 | down | chr19 | + | 7.88747 | 10.47398 | 4.50301 | 5.76098 | 6.67864 | 7.90800 | 8.15250 | 7.72588 | 6.75900 | 7.47080 | 8.28824 | 11.58146 | 8.05418 | 10.33289 | 11.57858 | 11.13507 | 11.63651 | 11.85053 | 11.91117 | 11.42672 |
| NM_052899 | GPRIN1 | 7.82082E-03 | 11.36854 | down | chr5 | - | 13.56871 | 6.60266 | 7.67790 | 7.50428 | 8.23098 | 14.16812 | 13.95228 | 14.13098 | 8.62224 | 14.23521 | 14.55529 | 14.59131 | 14.18350 | 12.46429 | 15.07199 | 16.23961 | 14.92926 | 14.63128 | 14.66663 | 15.62474 |
| ENST00000296682 | PRDM9 | 5.59244E-03 | 11.09584 | down | chr5 | + | 5.18130 | 12.08741 | 6.52448 | 6.11844 | 10.85520 | 8.32940 | 7.93951 | 7.85453 | 10.56844 | 4.55620 | 8.02097 | 13.79659 | 10.44095 | 7.65109 | 11.77037 | 7.23681 | 13.29585 | 11.97112 | 13.75053 | 13.36357 |
| uc001lcv.2 | DQ596646 | 9.44916E-08 | 10.98667 | down | chr10 | - | 2.32623 | 2.33005 | 2.33888 | 3.15776 | 2.32162 | 2.32238 | 2.32368 | 2.87343 | 2.32106 | 3.78967 | 2.83051 | 6.94312 | 7.64140 | 4.07524 | 6.26986 | 4.51336 | 7.27893 | 6.43668 | 6.45844 | 5.17643 |
| NM_001145054 | C2orf81 | 5.93000E-03 | 10.80877 | down | chr2 | - | 10.40962 | 5.27391 | 5.75379 | 6.25838 | 7.31852 | 6.72637 | 6.71962 | 7.09055 | 7.43252 | 10.48224 | 7.10220 | 13.59564 | 5.67709 | 8.49229 | 10.43409 | 6.60079 | 13.66677 | 11.45779 | 13.60380 | 13.29798 |
| ENST00000361227 | MT-ND3 | 1.96048E-04 | 10.55352 | down | chrM | + | 5.51213 | 6.57023 | 5.49655 | 5.36262 | 8.52591 | 4.67259 | 4.90643 | 7.90864 | 8.70033 | 8.00886 | 7.31263 | 10.62316 | 9.01643 | 7.67714 | 12.19204 | 7.57495 | 10.40179 | 12.63281 | 10.33198 | 9.85498 |
| NM_014346 | TBC1D22A | 4.52272E-03 | 10.47436 | down | chr22 | + | 7.37695 | 6.66231 | 8.85333 | 8.85162 | 11.09919 | 10.51716 | 10.65929 | 3.40405 | 11.09435 | 6.63938 | 3.46020 | 12.82671 | 11.50971 | 8.23098 | 10.87377 | 10.61099 | 12.64943 | 11.13507 | 12.87244 | 12.29550 |
| NM_018115 | SDAD1 | 1.95188E-02 | 10.35671 | down | chr4 | - | 2.58881 | 6.32314 | 2.33888 | 2.33029 | 3.25468 | 2.32238 | 2.32368 | 11.33113 | 3.51971 | 2.32208 | 11.47989 | 9.65960 | 8.10447 | 7.34533 | 7.58912 | 3.89141 | 9.64455 | 7.53694 | 9.61092 | 7.98939 |
| NM_001001664 | SPOPL | 7.81428E-04 | 10.35180 | down | chr2 | + | 6.34960 | 8.29036 | 7.08632 | 6.71873 | 6.89748 | 6.99534 | 6.36668 | 3.14250 | 6.42866 | 6.76662 | 4.24685 | 12.10045 | 10.27187 | 7.23681 | 7.64896 | 8.37750 | 12.17551 | 6.37708 | 12.15064 | 10.69858 |
| NM_002928 | RGS16 | 2.78226E-03 | 10.33625 | down | chr1 | - | 4.17867 | 7.13527 | 6.86555 | 6.47406 | 6.28082 | 5.17158 | 4.67885 | 2.92889 | 5.65483 | 2.88245 | 2.49881 | 11.63165 | 8.47210 | 5.91998 | 5.74957 | 5.94222 | 10.37360 | 5.32765 | 11.65775 | 10.04753 |
| ENST00000256257 | RNF122 | 1.65725E-06 | 10.07811 | down | chr8 | - | 4.84127 | 3.73258 | 2.33888 | 2.33029 | 2.32162 | 2.72620 | 2.47886 | 2.59621 | 2.32709 | 5.78331 | 2.49574 | 6.63779 | 7.44643 | 6.81991 | 5.37636 | 6.88495 | 7.52137 | 5.01904 | 6.37767 | 5.71019 |
| ENST00000361381 | MT-ND4 | 2.06174E-02 | 9.55254 | down | chrM | + | 6.60266 | 7.04556 | 5.94839 | 5.91175 | 4.22137 | 12.87103 | 12.95858 | 8.79543 | 4.59321 | 8.61456 | 8.61740 | 11.32984 | 9.59573 | 14.03489 | 6.95835 | 14.13098 | 11.61493 | 7.24046 | 11.07007 | 13.83856 |

**Table S5. Top 20 aberrantly down-regulated mRNAs in microarray analysis.** Seqname: sequence name. Gene symbol: gene name. P value: P value calculated from unpaired t-test. Fold change: the absolute ratio (no log scale) of normalized intensities between two groups (DCM vs ICM). Chr: chromosome number from which the mRNA is transcribed. Strand: the strand of chromosome from which the mRNA is transcribed; “+” is the sense strand of the chromosome, “−” is the antisense strand of the chromosome. DCM 1-11 and ICM1-9: normalized intensity of each sample (log2 transformed)
